# Similarity of the non-amyloid-β component and C-terminal tail of monomeric and tetrameric alpha-synuclein with 14-3-3 sigma

**DOI:** 10.1016/j.csbj.2021.09.011

**Published:** 2021-09-14

**Authors:** Sarah R. Evans, Colista West, Judith Klein-Seetharaman

**Affiliations:** a Colorado School of Mines, Quantitative Biosciences and Engineering, 1012 14^th^ St, Chemistry, Golden, CO 80401, USA; b Colorado School of Mines, Department of Chemistry, 1012 14^th^ St, Chemistry, Golden, CO 80401, USA

**Keywords:** αSyn, alpha-synuclein, pHSPB6, phosphorylated Heat Shock Protein beta-6, sHSP, small heat shock protein, SNCA, alpha-synuclein gene name, YWHAB, 14-3-3 protein beta isoform gene name, YWHAH, 14-3-3 protein eta isoform gene name, YWHAE, 14-3-3 protein epsilon isoform gene name, MAPT, microtubule-associated protein tau gene name, BAD, BCL2 associated agonist of cell death gene name, TH, tyrosine hydroxylase gene name, IDP, Intrinsically disorder protein(s), PPI, Protein-Protein interactions, SIP, shared interaction partner, Homology, Alpha-synuclein, Tetramer, 14-3-3 proteins, Protein structure, Prediction

## Abstract

Alpha-synuclein (αSyn) is often described as a predominantly disordered protein that has a propensity to self-assemble into toxic oligomers that are found in patients with Parkinson's and Alzheimer's diseases. αSyn's chaperone behavior and tetrameric structure are proposed to be protective against toxic oligomerization. In this paper, we extended the previously proposed similarity between αSyn and 14-3-3 proteins to the α-helical tetrameric species of αSyn in detail. 14-3-3 proteins are a family of well-folded proteins with seven human isoforms, and function in signal transduction and as molecular chaperones. We investigated protein homology, using sequence alignment, amyloid, and disorder prediction, as well as three-dimensional visualization and protein-interaction networks. Our results show sequence homology and structural similarity between the aggregation-prone non-amyloid-β component (NAC) residues Val-52 to Gly-111 in αSyn and 14-3-3 sigma residues Leu-12 to Gly-78. We identified an additional region of sequence homology in the C-terminal region of αSyn (residues Ser-129 to Asp-135) and a C-terminal loop of 14-3-3 between helix αH and αI (residues Ser-209 to Asp-215). This data indicates αSyn shares conserved domain architecture with small heat shock proteins. We show predicted regions of high amyloidogenic propensity and intrinsic structural disorder in αSyn coincide with amyloidogenic and disordered predictions for 14-3-3 proteins. The homology in the NAC region aligns with residues involved in dimer- and tetramerization of the non-amyloidogenic 14-3-3 proteins. Because 14-3-3 proteins are generally not prone to misfolding, our results lend further support to the hypothesis that the NAC region is critical to the assembly of αSyn into the non-toxic tetrameric state.

## Graphical abstract

**Figure:**
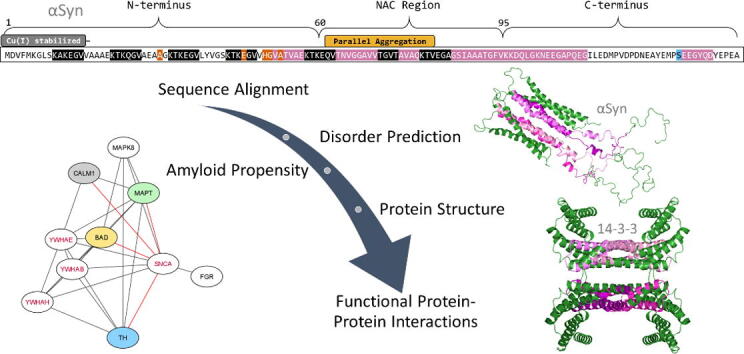

## 1 Introduction

Parkinson's Disease (PD) is a multifactorial neurodegenerative disease characterized by the toxic aggregation of the amyloidogenic protein alpha-synuclein (αSyn) [1], [2]. Current therapeutic solutions are limited to the treatment of existing symptoms using complicated pharmacological or invasive surgical strategies. Notably, a joint Parkinson's Foundation and Michael J. Fox Foundation-funded study indicates the 2017 economic burden of PD is nearly $52 billion in the United States alone, and projects this cost to continue increasing [3]. The largest contribution to this value is medical costs, which has led researchers to consider the non-toxic tetrameric species of αSyn as a therapeutic solution [4], [5], [6]. The primary focus of research on PD is largely focused on the neurotoxic effects of αSyn [1], [5], [7], however, preventative therapeutics will necessarily rely on understanding the normal functions of αSyn. Understanding the αSyn tetramer and ordered transition states related to αSyn's chaperone behavior may be useful to mechanistic drug design for synucleinopathies since these species of αSyn are resistant to toxic oligomerization.

Although there is consensus on the involvement of αSyn in the pathogenesis of PD, the soluble structure of αSyn and its function are still not completely understood. The proposed functional role of αSyn has a broad range including acting as a chaperone [1], [8], [9], [10], [11], a chemotactic protein [12], [13], and a tether for synaptic vesicles [14]. αSyn is concentrated at synaptic terminals and is commonly accepted to be involved in facilitating neurotransmission through synaptic vesicle transport and regulation [1], [15]. However, the experimental evidence of αSyn's chaperone behavior suggests only an indirect neuroprotective effect on the co-chaperone, cysteine string protein (CSPα) [16]. The misfolded form of αSyn is consistently identified as a key component of the Lewy body inclusions in neurons that are a hallmark of patients with PD and other synucleopathies [1]. The ability to form oligomeric species of αSyn is cited as a key feature of both its pathogenesis in PD as well as its proposed ability to act as a ferrireductase [7], [17], [18]. The point mutations identified for αSyn (Ala-30-Pro, Glu-46-Lys, His-50-Gln, Gly-51-Asp, and Ala-53-Thr) are associated with familial PD; these mutations have been shown to modulate the propensity for aggregation [2], [5], [19]. There is also evidence of the stable tetrameric structure in human erythrocytes and neurons [4], [20], [21], and the tetramer is proposed to exist in metastable equilibrium with the unfolded monomer [6]. Thus, while evidence suggests the disordered monomer is the predominant species physiologically [22], [23], αSyn also transitions to physiologically relevant ordered states that arise from binding interactions [20], [24].

**Fig. 1:**
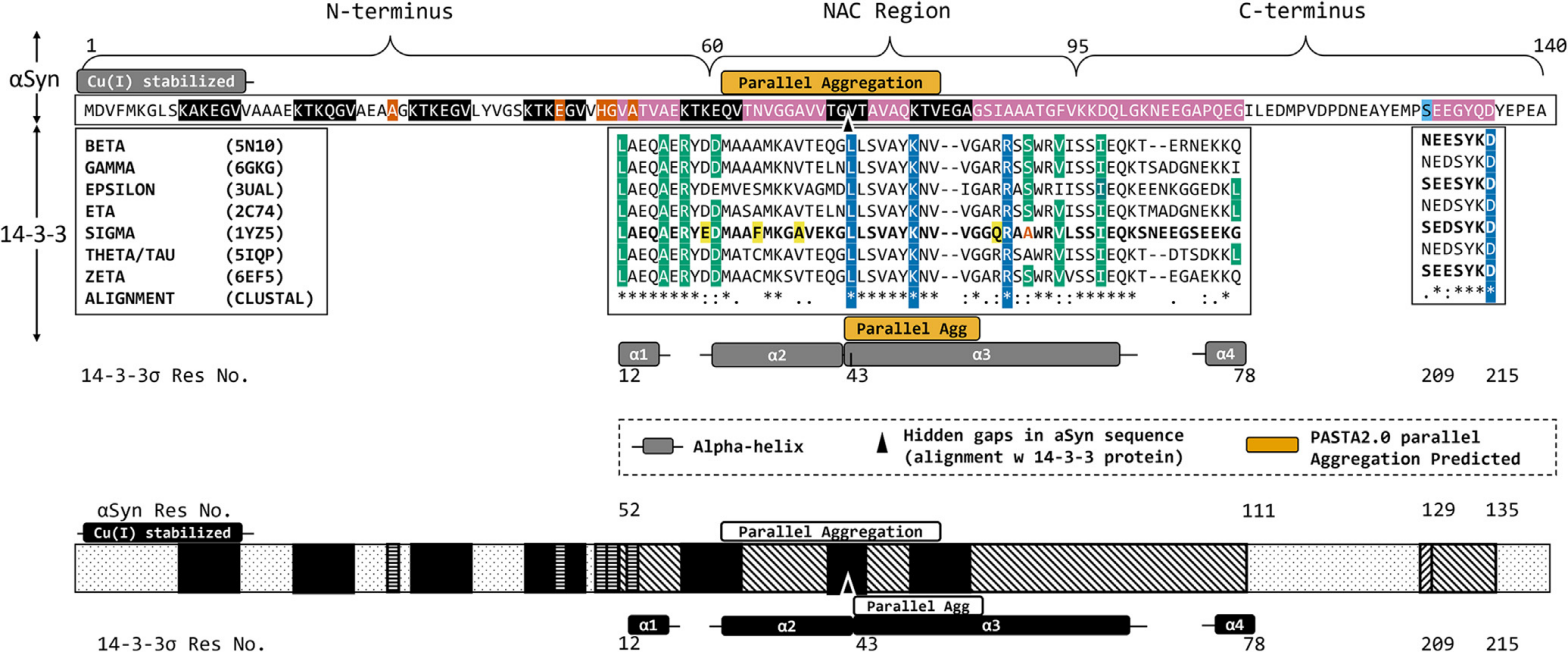
Sequence alignment between αSyn and 14-3-3 protein. Top to bottom: N-terminus, NAC, and C-terminal brackets indicate the amino acids contained in each respective region of αSyn, labeled with αSyn residue number. The grey bar is over αSyn residues that are stabilized by Cu(I)/Cu(II) binding. The light orange bar indicates residues that are predicted to have high propensity for aggregation. The αSyn sequence is colored as follows: seven imperfect lipid binding motifs (black highlight with white letters); familial point mutations (dark orange with white letter); sequence alignment with 14-3-3σ (pink with white leters); phosphorylated Ser-129 (light blue with black letter). The black triangle points at the location of the hidden gaps in the αSyn alignment corresponding to nine residues in the 14-3-3σ sequence (the complete BLAST alignment output is shown in the Supplemental Information). Under the αSyn alignment is the alignment region identified for 14-3-3σ. All seven isoforms are labeled to the left of the sequence segment. The 14-3-3 sequence shows the homodimer contact residues (teal highlight with white letters), and the amphipathic groove contacts (dark blue highlight with white letters). Yellow highlighted residues show unique residues in the 14-3-3σ isoform. The 14-3-3σ residue Ala-58 is shown as an orange letter. 14-3-3 alpha-helices or portions of helices that overlap with the alignment segment are matched to the 14-3-3 sequence. Relevant residue numbers are shown below the diagram for 14-3-3σ and αSyn respectively. (For interpretation of the references to colour in this figure legend, the reader is referred to the web version of this article.)

The αSyn monomer (Fig. 1) is composed of 140 amino acids with three characteristic regions: An N-terminal α-helical region (res. Met-1 to Thr-60), the amphipathic non-amyloid-β component (NAC) region (res. Lys-61 to Val-95), and the flexible, acidic C-terminal region (res. Lys-96 to Ala-140). Traditional biophysical techniques are limited in their use for structural determination of αSyn because it exists primarily as an intrinsically disordered protein (IDP) [6], [20], [22], [23], [24], [25]. IDPs lack a three-dimensional (3D) structure from which to infer function, and αSyn likely exists as a dynamic protein that transitions between multiple metastable states existing in equilibrium [1], [6], [20], [26]. The complexity of the biophysical characterization of αSyn is detailed by Alderson and Markley [24]. One proposed state of αSyn is the tetrameric species, which was successfully purified and measured experimentally using Nuclear Magnetic Resonance spectroscopy; however, the resulting tetramer used a modified monomer with an additional 11-mer peptide [21]. This structural model and the micelle-bound monomer [27] was used in multiple computational predictions of the tetramer [28], [29], [30]. Model predictions indicate the tetrameric αSyn structure is stabilized by the hydrophobic NAC core residues, and the characteristic hairpin turn separates the two α-helices (Fig. 1) [21], [27], [28], [29], [30]. Tetrameric αSyn presents as a metastable α-helically structured multimer that resists aggregation [6], [20], [31]. However, the prevalence and stability of the tetramer are low [20], [21], [22], [31], and the normal molecular mechanisms of the formation of the tetrameric αSyn are still uncertain. In addition, the metastable state of αSyn leads to complications in investigating the native state of αSyn using standard purification protocols [20], highlighting the need for computational and structure prediction techniques. Still, exact loop and helix structures and core residue interactions of disordered proteins should be interpreted with the knowledge that such predictions impart the structural features of the template model onto the prediction model. Homology analysis starting from basic sequence alignment may be an ideal way to analyze IDPs to avoid prematurely imposing structural constraints. Sequence alignment was used to predict remote homology between αSyn and 14-3-3 proteins reported by Ostrerova *et al.* in 1999.

14-3-3 proteins are a family of adapter proteins that are structurally well-understood, with seven highly conserved isoforms identified in humans (beta, β; eta, η; zeta/delta, ζ/δ; sigma, σ; gamma, γ; epsilon, ε; theta/tau, θ/τ) [32]. They mediate signal transduction by binding to proteins containing a phosphoserine/phosphothreonine recognition motif [33], [34]. Each monomer contains nine α-helices, where the canonical dimer and tetramer contact residues are between helices αA, αB, αC, and αD, forming a cup-shaped structure [32], [34]. 14-3-3 proteins are known to form homo- and heterodimers and tetramers [32], [34]. A substantial number of crystal structures [35], [36], [37], [38], [39], [40], [41] and co-crystal structures [42], [43], [44] are available in the Research Collaboratory for Structural Bioinformatics protein data bank (RCSB PDB). 14-3-3 proteins exhibit an ATP-dependent response to heat shock conditions and have been described experimentally to have chaperone function [45], including for the Raf kinase family [9], [34], [36], [42], and Exotoxins S and T [44]. Finally, they even interact with another class of chaperones, small heat shock proteins (sHSP), one of which was co-crystallized with 14-3-3σ stabilizing intrinsically disordered regions of the sHSP [43].

Ostrerova *et al*. identified two regions of sequence homology between αSyn (αSyn residues Leu-8 to Glu-61) and 14-3-3 isoforms β, ζ/δ, τ, and ε (14-3-3 residues Leu-45 to Leu-102). αSyn chaperone behavior has since been linked to the HSP family [8], [10]. The α-helical secondary structure of 14-3-3 proteins may correspond with the ordered, α-helical N-terminus of metal- and lipid bound αSyn [27], [46], [47]. Notably, phosphorylated Ser-129 in the C-terminus of αSyn was shown to preferentially interact with 14-3-3 ε, γ, and ζ/δ isoforms over non-phosphorylated αSyn [48]. In PD-affected patients, αSyn is predominantly phosphorylated at Ser-129, which may alter αSyn PPIs [49]. Furthermore, a recent study demonstrates 14-3-3σ colocalizes with αSyn in Lewy bodies from human brain tissue, indicating 14-3-3 participation in amyloidogenesis [50]. Despite these connections, there are limited studies that show 14-3-3 is a homolog of αSyn, and existing evidence of αSyn's chaperone behavior still refer back to the original sequence-based predictions reported by Ostrerova *et al*. in 1999.

Significant work was done to understand 14-3-3 proteins and αSyn in greater detail since this study; however, the homology between αSyn and 14-3-3 has not been reevaluated with new data in mind. The combination of homology analysis and computational structure prediction techniques presents a valuable avenue for understanding the key structural components of tetrameric αSyn. In this paper we investigated the structural basis for the stability of the tetrameric species of αSyn using multiple computational and prediction techniques that instead originate from sequence similarity. We built upon the initial results by Ostrerova *et al.* to further investigate αSyn's similarities to chaperone proteins. We hypothesized the tetrameric species of αSyn will also show similarity to 14-3-3 proteins, and used the sequence homology results to inform the structural analysis of αSyn's ordered states. We used the Basic Local Alignment Search Tool (BLAST) to identify isoform specific sequence homology, we included the full sequences of both protein families and discussed all seven isoform alignments. We propose our homology analysis will elucidate structurally and functionally inherited features of αSyn's ordered states through comparison to the evolutionarily conserved 14-3-3 family.

## 2 Methods

### 2.1 BLAST sequence alignment

All sequences were submitted to protein BLAST using the FASTA sequence download from their respective RCSB PDB entries. The sequence input for all proteins begins with the first MET residue for consistency. All regions identified by the BLAST output were considered in the analysis. We use the computational alignment prediction program, BLAST, to determine the alignment regions for each isoform separately. We did not use the MSA function; the individual (one to one) alignment means we are not restricting the alignment to yield results common to all eight sequences. We considered each of the statistical output values reported by BLAST in the analysis. BLAST determines the most similar local alignment between sequences, which we use as a tool to infer to what degree alpha-synuclein is related to 14-3-3 relative to each of the seven human isoforms. In this analysis we consider significant percent identity to be above the standard threshold value of 30% and high percent identity to be greater than 50%. We used alignment length and bit score as a predictor of significance over percent identity, which allowed us to analyze the proteins under the combined lens of sequence and functional homology. The bit scores for all alignments in this analysis are less than 50, which is the common threshold indicator for significance. We instead perform an internal comparison of the bit scores to make a relative determination because homology has been previously established. All subsequent alignments were made with respect to the αSyn/14-3-3σ isoform to simplify the data interpretation. We account for insertions/deletions that may have a significant impact to the alignment in the discussion. We include the τ/θ isoform results, which did not return any significant alignment to αSyn. Alignment regions between αSyn and 14-3-3 that show repeats across more than 50% of the 14-3-3 isoforms, such as with the C-terminus of αSyn, were confirmed to be aligned using Clustal Omega MSA on the 14-3-3 isoforms. These regions were considered as a ubiquitous alignment region for all 14-3-3 isoforms since they have a greater than 50% percent identity score and >80% percent positives.

### 2.2 Clustal Omega 14-3-3 isoform alignment and synuclein family alignment

Clustal Omega EBI web server (https://www.ebi.ac.uk/Tools/msa/clustalo/) was used to determine the full-sequence multiple sequence alignments for each family of proteins respectively. Clustal Omega multiple sequence alignment has better performance for closely related sequences than BLAST. The protein FASTA sequences used in the BLAST analysis were submitted to the Multiple Sequence Alignment (MSA) tool. The sequence input for all proteins begins with the first MET residue for consistency. One file, containing the FASTA sequences of all seven 14-3-3 isoforms, was submitted in a query for the 14-3-3 isoforms. A separate file containing the FASTA sequences for alpha, beta, and gamma synuclein was submitted for the synuclein family. The default settings were used for the output format. The 14-3-3 isoform multiple sequence alignment was used to identify the conserved regions of the 14-3-3 family, and to determine the residues that correspond to the σ isoform BLAST alignment with αSyn. Highly conserved regions are used to support the extension of sequence homology across all isoforms in the 14-3-3 family. Clustal Omega uses the “ * ” symbol to indicate identical conserved residues, “ : ” to indicates the alignment has strongly similar residues, the “ . ” indicates the residues have weakly similar properties, and no symbol is for residues that are not conserved. Further details on the Clustal Omega data interpretation can be found in the website documentation. Results are included in the supplemental information file in Figs. S1.2 and S1.3.

### 2.3 PONDR result alignments and disorder comparison

Full FASTA sequences starting with the first MET residue for each protein were submitted to the Predictors of Natural Disordered Regions (PONDR) web server (http://pondr.com/). In all cases, the VL-XT predictions are shown, which represent the merger of predictions trained on disordered regions characterized by different approaches [51]. The PONDR results were considered with respect to the αSyn/14-3-3σ isoform sequence alignment for consistency. The PONDR results were gathered individually by submitting the FASTA sequence one at a time to the PONDR prediction; the FASTA files are the same as those used for the protein BLAST and Clustal Omega alignments. The raw data for the predictor values was copied from the PONDR website results page, and evaluated against the BLAST alignment results. The sequence alignments across the 14-3-3 isoforms were matched to the Clustal Omega Multiple Sequence Alignment results described above [52], [53]. We identified the alignments for 14-3-3σ residues Leu-12 to Gly-78 and Ser-209 to Asp-215 (Table 2) corresponding to all 14-3-3 isoforms. Then the PONDR results for each 14-3-3 isoform was aligned to the regions in αSyn identified by the BLAST alignment to the 14-3-3σ isoform. We used these alignments to compare 14-3-3 PONDR scores to the αSyn PONDR score. The scores for beta and gamma synuclein were gathered individually and aligned to αSyn according to the Clustal Omega alignment results for consistency. The PONDR scores were visually similar, thus were averaged by residue across all seven 14-3-3 human isoforms based on the Clustal Omega alignment results.

**Table 1:** PASTA2.0 Methods, prediction energy outputs. The best energy for each sequence is presented in the table as a summary of the PASTA 2.0 direct output from the webserver.

Protein name (from fasta header)	length	# amyloids	best energy	% disorder	% α-helix	% β-strand	% coil
1XQ8_Alphasynuclein	140	20	−7.24	34.28	26.43	22.14	51.43
5N10_1433-BETA	246	7	−5.43	17.88	76.02	0	23.98
6GKG_1433-GAMMA	234	3	−5.43	6.84	78.21	1.71	20.09
3UAL_1433-EPSILON	232	9	−5.40	5.60	81.03	0.43	18.53
2C74_1433-ETA	246	4	−5.43	12.19	79.67	0	20.33
1YZ5_1433-SIGMA	248	2	−5.43	20.16	73.79	0	26.21
5IQP_1433-THETATAU	245	9	−5.43	11.02	77.14	0	22.86
6EF5_1433-ZETADELTA	245	7	−5.43	12.65	79.59	0	20.41

**Table 2:** BLAST sequence alignment results for query protein, αSyn, and subject protein, 14-3-3 human isoforms. Results are in order of highest bit score (to αSyn) for each predicted region. Each 14-3-3 isoform was aligned individually to αSyn to avoid missing homologous regions. The dashes under the Ostrerova lab results indicate the value is not calculated. The theta(tau) isoform BLAST alignment did not yield significant results.

Isoform	Subject (14-3-3)	bit score	% identity	alignment length	gap opens	q. start	q. end	s. start	s. end	e value	% positives
sigma	1YZ5	16.9	31.67	69	11	52	111	12	78	0.19	44.93
Ostrerova et al, 1999	**beta, zeta, theta, epsilon**	–	**36**–**43**	**53**	**–**	**8**	**61**	**45**	**102**	**–**	**–**
epsilon	3UAL	15.8	71.43	7	0	129	135	210	216	0.44	85.71
zeta(delta)	1A4O	15.4	71.43	7	0	129	135	207	213	4.2	85.71
zeta(delta)	6EF5	15.4	71.43	7	0	129	135	207	213	0.6	85.71
beta	5 N10	14.2	57.14	7	0	129	135	209	215	1.6	85.71
zeta	6EF5	14.2	50.00	10	0	29	38	133	142	1.6	80.00
sigma	1YZ5	13.9	57.14	7	0	129	135	209	215	1.7	85.71
gamma	6GKG	13.9	30.43	23	0	94	116	1	23	1.8	47.83
eta	2C74	12.7	30.00	20	0	97	116	4	23	4.9	50.00
sigma	1YZ5	11.9	45.45	11	0	129	139	70	80	9.1	72.73
theta(tau)	5IQP	Not Significant									

### 2.4 PASTA 2.0 aggregation predictions

The same FASTA protein sequences were submitted to the PASTA 2.0 online server (http://protein.bio.unipd.it/pasta2/) under the regular input. The default options settings were determined to be sufficient for this analysis: Custom thresholds (top pairings and energy are used), up to 20 top pairing energies, an energy threshold of −5, 1.0 Pasta Energy Unit = 1.192 Kcal/mol, true positive rate (TPR) sensitivity of 40.5%, false positive rate (FPR) (1-specificity) of 4.7%. The best energy for each sequence was presented in Table 1 as a summary of the PASTA 2.0 output. The raw data output for the Aggregation Probability was extracted from the output data set into a text file. The probability values are given by residue number, so the results are manually matched to the alignment results from Clustal Omega to ensure the correct correspondence to the residue letters and multiple sequence comparison of the 14-3-3 isoforms. The aggregation probability alignments to the αSyn sequence use the BLAST alignment results for 14-3-3σ (Supplemental Fig. S1.2).

### 2.5 AMYLPRED2 aggregation predictions

Full FASTA protein sequences were submitted to the AMYLPRED2 web tool (http://biophysics.biol.uoa.gr/AMYLPRED2) for one chain of each protein. Each sequence was submitted separately for αSyn and for each of the seven 14-3-3 isoforms (eight submission files total). Ten amyloid prediction methods were scanned, and consensus results were reported for interpretation. Only regions with results greater than Consensus 5 for αSyn and Consensus 5 for 14-3-3 were considered to have a high propensity for amyloid formation. This consensus threshold is well within the suggested balance. AMYLPRED2 empirically determines the best balance for determining consensus to be at least n/2 of n selected methods [54]. Consensus 5 results were determined to be sufficient for αSyn comparison to 14-3-3 isoforms as a result of the strong correspondence to the PASTA2.0 disorder predictions. 14-3-3ζ/δ was used as representative for all seven isoforms.

### 2.6 STRING interactions predictions

The list of interactors for 14-3-3 proteins (all isoforms) and αSyn (along with the paralogs gamma- and beta-synuclein) were extracted from the Human Protein Reference Database (http://hprd.org/index_html). We did not modify the contents (by adding missing interactions) of the databases used in this analysis. Each protein and isoform were queried separately. The lists of interactors for all seven human isoforms of 14-3-3 (14-3-3x) were combined into a master list, and the duplicate interactions were removed to prevent unanticipated effects from duplicates. Similarly, alpha-, beta-, and gamma-synuclein (α, β, γSyn respectively) interactors were combined to create a master list. The 14-3-3 and synuclein family protein interactions were submitted to the STRING database search “Multiple Proteins by Names/Identifiers (https://string-db.org/) as two separate queries, with *Homo Sapiens* selected in the “Organism” field. The string interaction results for 14-3-3x were downloaded and manually searched for common interaction node-pairs against the synuclein download results to identify interactions that were common to both protein networks. All results were compared, and only the resultant node-pairs showing greater than 0.4 combined confidence score was considered in the analysis. This lower bound ensures we only consider interactions that return a combined score of medium confidence or better.

### 2.7 PyMOL residue identification for dimer and tetramer contacts in 14-3-3σ

The 3D structure details of 14-3-3σ were investigated using the molecular visualization system, PyMOL (https://pymol.org/2/). The visualization was performed using the PDB Format file for each protein downloaded from the RCSB PDB (https://www.rcsb.org/). Dimer and tetramer contacts were identified between the alignment residues and any other residues in the protein that are within 5.0 Å of the central homologous region corresponding to the NAC region of αSyn (14-3-3σ residues Leu-12 to Gly-78). The Van der Waals contact distance (approximately 5.0 Å) was chosen as an upper bound to ensure our analysis does not include any indirect interactions. First, all residues within 5.0 Å of the homologous residues were highlighted; the selection thus includes neighboring residues in order to include inter- and intrachain contacts. Next, polar contacts with a cutoff of 3.0 Å were found for residues within that selection. We implement a tighter bound of 3.0 Å (compared to the more accepted bound of 3.5 Å for hydrogen bond distances) to restrict the contacts to within a range equal to the average bond length of a hydrogen bond. This bound still allowed us to focus on the interactions within our area of interest and the strongest contributors. Contact residues were then manually selected for contacts found between different chains. Informative contact distances were measured using the PyMOL measurement tool. Tetramer contacts for the 14-3-3ζ/δ crystal structure were similarly identified, first by finding contacts within 5.0 Å of the alignment region.

### 2.8 PHYLIP phylogenetic tree using the protein sequence parsimony method

We used the PHYLIP, PHYLogeny Inference Package (https://evolution.gs.washington.edu/phylip.html), to generate a phylogenetic tree for the BLAST sequence alignment shown in Fig. 1. We first created the input file starting with the Clustal Omega MSA result for the synuclein family. Next, we add the 14-3-3 sigma BLAST sequence alignment segment shown in Table 1 and Fig. 1. The other six human isoforms are then aligned to the sigma isoform regions according to the Clustal Omega MSA result for the 14-3-3 family. Gaps from the 14-3-3 sigma BLAST alignment and gaps from β- and γSyn MSA were carried through the 14-3-3 human isoforms as “-” symbols. Regions of unknown alignment between 14 and 3-3σ and αSyn are indicated as “?” according to the PHYLIP documentation. The combined alignment was used as the input file. We show the consensus tree from the Protpars, Protein Sequence Parsimony Method, which infers unrooted phylogeny from protein sequences.

## 3 Results

### 3.1 Sequence alignment within the N-terminus and C-terminus.

To investigate the conserved amino acids of αSyn, we evaluated sequence alignment for each of the seven human isoforms of 14-3-3 using BLAST. The first consideration we made was the functional variation of the 14-3-3 human isoforms despite the observed high sequence identity [34]. In this work we ultimately focused on the significance of the functional similarity between 14 and 3-3 and αSyn, thus each 14-3-3 isoform sequence was compared individually to αSyn. The BLAST sequence alignment results indicate two distinct regions of homology: one region within the NAC, aggregation-prone region of αSyn, and the second at the C-terminus of αSyn. These results are summarized in Fig. 1 and shown in the context of known features of the αSyn protein sequence. Our 14-3-3σ alignment results share overlap with the 14-3-3 alignment region identified by Ostrerova et al. The researchers identified two regions of sequence homology between αSyn (αSyn residues Leu-8 to Glu-61) and 14-3-3 isoforms β, ζ/δ, τ/θ, and ε (14-3-3σ residues Ser-45 to Leu-102). The corresponding αSyn residues in row 1 cover the amyloidogenic NAC region and indicate an overlap of 10 amino acids with the Ostrerova *et al*. results. The corresponding 14-3-3 alignment segment in row 1 shares a 34-amino acid overlap with the alignment by Ostrerova et al. (Table 2).

The αSyn residues Ser-129 to Asp-135 show homology to four 14-3-3 isoforms (ζ/δ, ε, σ, β) as well as to the 14-3-3CD loop, a loop region of 14-3-3 between αC and αD (Table 2 and Fig. 1). Our results indicate the highest percent identity is contained within the c-terminal region of αSyn and a c-terminal loop of 14-3-3 between helix αH and αI (HI loop). The short seven-amino-acid alignment, from αSyn Ser-129 to Asp-135, has a high percent identity (>70%) that was consistent across multiple isoform alignments. This was unlike the other segments which show αSyn alignment with only one or two isoforms. The sequence homology predicted between residues Ser-129 to Asp135 in αSyn aligns with residues Ser-209 to Asp-215 in the HI loop of the 14-3-3 family. Furthermore, BLAST identifies a long segment (69 residues) of sequence homology between αSyn residues Val-52 to Gly-111 and 14-3-3σ residues Leu-12 to Gly-78 as shown in Table 2. The 14-3-3σ isoform shows one of the lowest percent identity scores, however it contains the longest region of homology, and the highest bit score. This segment corresponds to the highly conserved amphipathic binding groove in the 14-3-3 family [34]. Our alignment includes three highly conserved 14-3-3 binding residues (Leu-43, Lys-49, Arg-56) as well as the isoform-specific CD loop, and the dimerization contact residues Arg-18, Lys-74, and Asp-21. Notably, this domain architecture is reminiscent of sHSPs [55].

We illustrate the results with respect to an unbroken αSyn sequence. The BLAST protein alignment shows nine gaps between Gly-73 and Val-74 in the αSyn sequence compared to the 14-3-3σ sequence (Supplemental Fig. S1.2). The nine amino acid insertions in 14-3-3σ that correspond to the alignment gaps in αSyn between Gly-73 and Val-74 are hidden during the analysis in order to view the similarities with respect to the αSyn sequence. This allows us to illustrate the sequence, disorder, and amyloid propensity similarities more clearly, but does not significantly impact the interpretation of the results. Additionally, there are two gaps in the 14-3-3σ corresponding to αSyn residues Val-51 and Val-52 (Fig. S1.2). This short, two-amino acid insertion was identified to be in the middle of the αC helix of 14-3-3 proteins. ζ/δ

### 3.2 Predicted disorder within the BLAST sequence alignments.

**Fig. 2:**
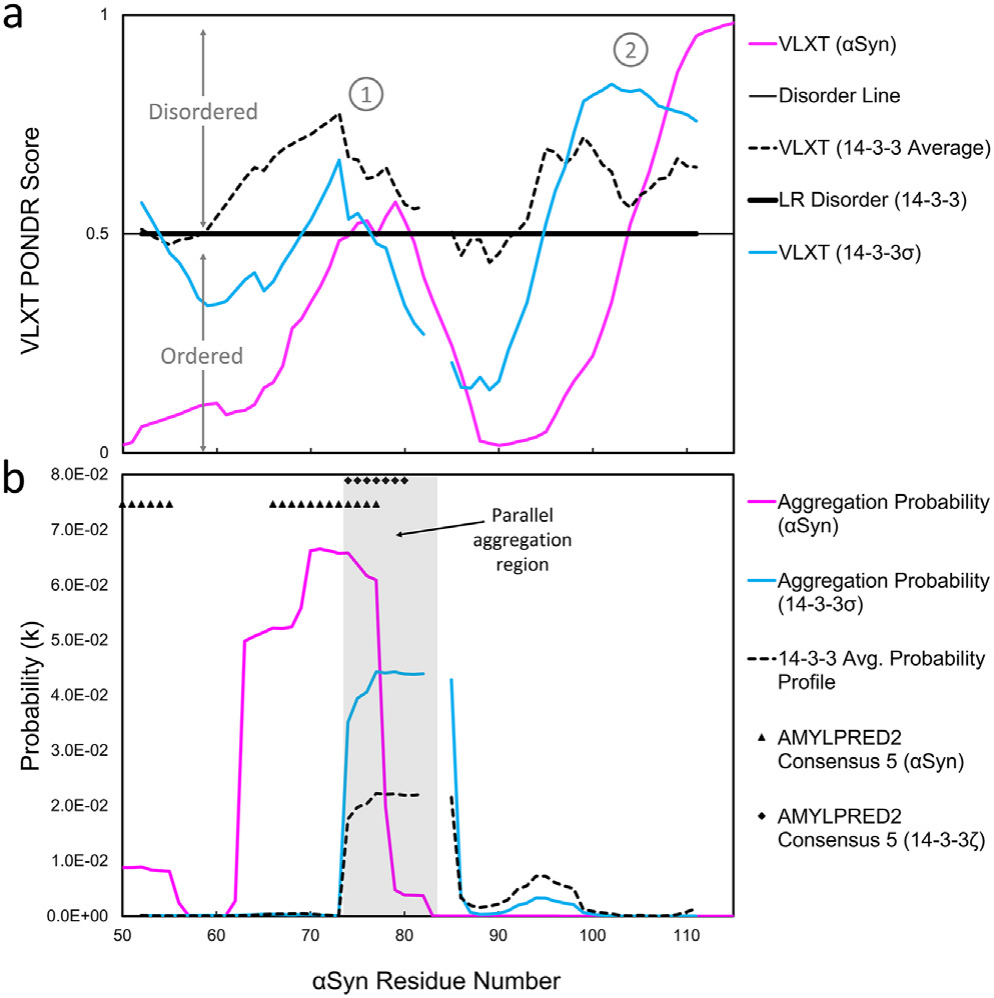
Disorder and Amyloid predictions for αSyn overlaid with the 14-3-3 protein results. (a) The VL-XT probability value is displayed on the y-axis plotted against the αSyn amino acid number on the x-axis. The disorder line is displayed at y-axis value of 0.5 (solid black line); the protein is predicted to be disordered for data above the line and ordered for data below the line. The VL-XT results for αSyn (solid magenta line), 14-3-3 sigma (solid blue line), and the averaged VL-XT data for all seven 14-3-3 isoforms (black dashed line) are shown with the αSyn amino acid number on the x-axis. The predicted long-range disorder for 14-3-3 is included as a thick solid black line overlaid with the disorder line at y = 0.5. Two distinct peak regions are indicated by the circled numbers 1 and 2 respectively. (b) The PASTA2.0 aggregation probability [k] results are shown on the y-axis plotted against the αSyn amino acid number on the x-axis; αSyn (solid magenta line), 14-3-3 sigma (solid blue line), and the averaged probability data for all seven 14-3-3 isoforms (black dashed line). PASTA 2.0 predicts parallel aggregation for αSyn and 14-3-3 proteins. The common region between residues Glu-72 and Arg-82 is highlighted using a shaded grey box and labeled. The sequence alignment region of 14-3-3 is displayed against the corresponding αSyn residues Val-52 to Gly-111 and Ser-129 to Ala-135 predicted by BLAST. The AMYLPRED2 Consensus 5 predictions have no numerical value and are shown using a secondary axis above the PASTA 2.0 results: Consensus 5 for αSyn (black triangles) and the Consensus 5 region for 14-3-3 zeta (black squares). (For interpretation of the references to colour in this figure legend, the reader is referred to the web version of this article.)

Next we extended the bioinformatics comparison between αSyn and 14-3-3 beyond sequence alignment methods with disorder and amyloid propensity analysis. The disorder and amyloidogenic properties of αSyn are characteristic of the pathogenic misfolding found in neurodegeneration. This data may also be relevant to the aggregation behavior of 14-3-3 proteins related to Lewy bodies and Lewy body neurites. We first used PONDR to determine the predicted structural disorder of αSyn compared to 14-3-3. Fig. 2a shows the PONDR results for αSyn compared against the Clustal Omega MSA of the 14-3-3 isoforms in the NAC alignment region. We again illustrate the data without the gap segment in the αSyn sequence and corresponding insertions in 14-3-3 between residues Gly-73 and Val-74 for clarity. The discontinuity in the graph are the two gaps in the αC helix of 14-3-3. The averaged PONDR score for the seven 14-3-3 isoforms showed two distinct peaks that correspond to the two peaks seen in αSyn indicating a higher prediction of disorder for these regions. The PONDR predictions indicated similarity in the disorder propensity of the alignment residues found between αSyn and 14-3-3 ranging from αSyn residues Val-52 to Gly-111. A more detailed version of this graph can be viewed in the supplemental information (Fig. S2.1).

**Fig. 3:**
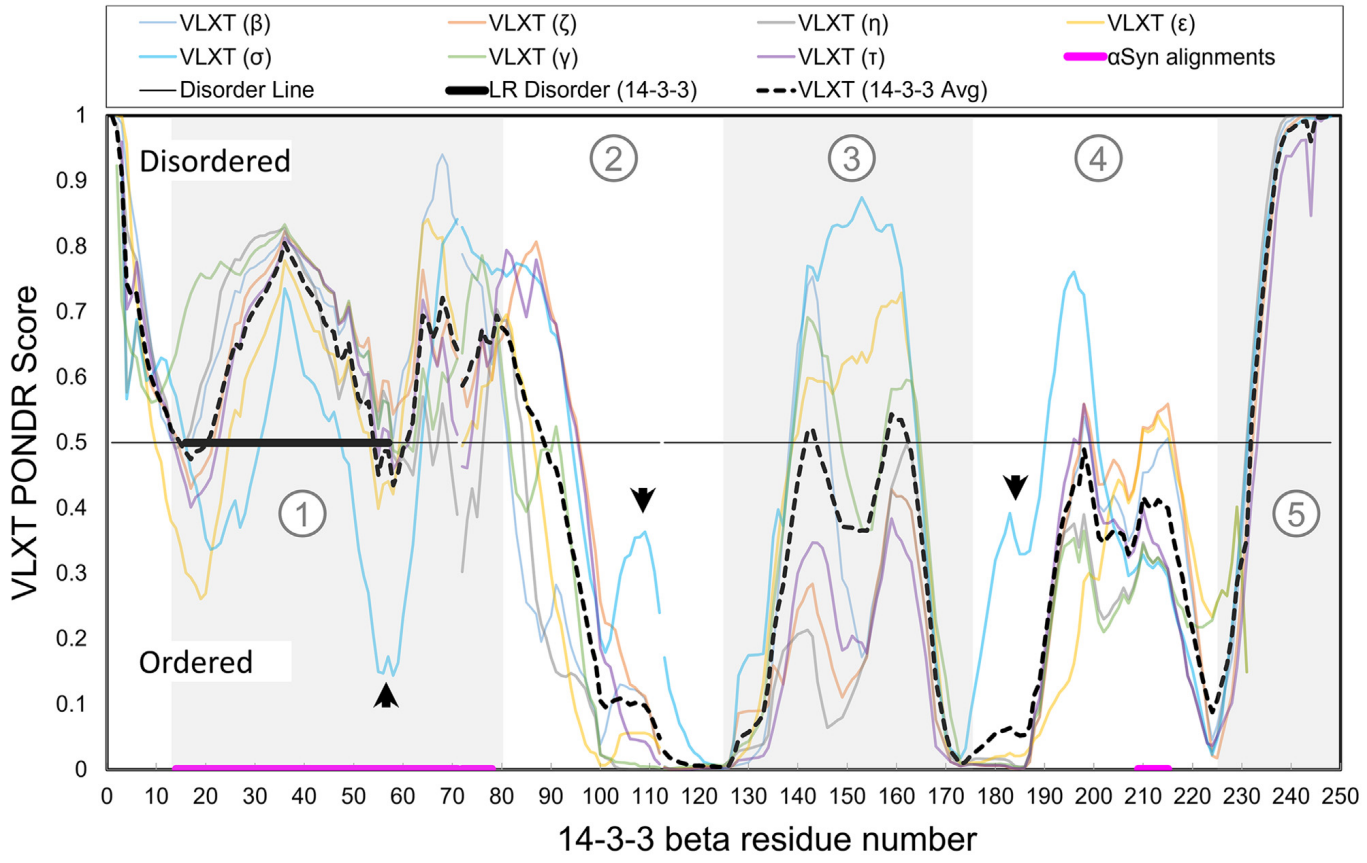
Overlay of all seven 14-3-3 isoform PONDR predictions (full sequence). The VLXT probability value is displayed on the y-axis plotted against the 14-3-3 beta isoform amino acid number on the x-axis. The disorder line is displayed at y = 0.5 (solid black line); the protein is predicted to be disordered for data above the line and ordered for data below the line (labeled). The averaged data for the seven 14-3-3 isoforms is graphed as a black dashed line. Long-range disorder is shown as a thick black line to indicate the common residues to the β, γ, η, and ζ results (centered at PONDER Score y = 0.5). Five distinct regions are identified with the numbers 1 through 5 from left to right with alternating shading. Region 1 contains residues from approximately Lys-12 to Met-80; Region 2 contains residues Gly-81 to Gly-125; Region 3 contains residues from Asp-126 to Asn-175; Region 4 contains residues from Phe-176 to Asp-225; Region 5 contains residues from Asn-226 on. Three black arrowheads mark distinguishing features of the 14-3-3 sigma results. The sequence alignment between αSyn's non-amyloid beta region and C-terminus is represented at the base of the graph as a thick magenta line. The legend is shown at the top of the graph. (For interpretation of the references to colour in this figure legend, the reader is referred to the web version of this article.)

Of the seven isoforms, it was visually apparent that the 14-3-3σ isoform had the highest degree of overlap to αSyn. Fig. 3 shows the PONDR data for all seven 14-3-3 isoforms and a magenta line corresponding to αSyn residues Val-52 to Gly-111 (see Fig. 1). Specifically we point out residues Ala-57 to Arg-60 as distinct among the 14-3-3 isoforms in region 1, and corresponds to the sequence alignment to αSyn. We individually inspected the PONDR profiles of the remaining seven isoforms and found regions of long-range disorder in 14-3-3 isoforms β, γ, η, and ζ/δ within the first 100 residues. We show only the long-range disorder for the η isoform since this region is shared among the results for β, γ, η, and ζ/δ (Fig. 3). The long-range disorder prediction covers the first four alpha-helical structures of 14-3-3 proteins as was well-documented through crystallography experiments [36], [37], [43], [44], [45]. Long-range disorder was not predicted for αSyn in this alignment segment, however the βSyn and γSyn results predict a combined long range disorder corresponding to αSyn residues Val-70 to Ala-140 (Fig. S2.2).

The long range disorder predicted in the 14-3-3 isoforms corresponds to the long-range disorder predicted for βSyn and γSyn (Fig. 3 and Fig. S2.2). We use the αSyn paralogs, βSyn and γSyn, to point out that the synuclein family is highly conserved in the N-terminus, therefore are useful for interpreting similarities between the 14-3-3 family of proteins. The PONDR scores for γSyn and βSyn are also predicted to have some disorder, especially near αSyn alignment residues Val-52 to Val-82 and Ala-85 to Leu-100. Yet, the disorder prediction results do not show strong overlap in the C-terminus alignment region of αSyn and the HI loop of 14-3-3, even for 14-3-3σ. This result from 14-3-3 is distinct from the strong prediction of disorder for α-, β-, and γSyn in the same alignment region. These 14-3-3 residues (corresponding to 14-3-3σ Ser-209 to Asp-215) are frequently a low-density region in 14-3-3 crystal structures, and we expected this region to have a higher PONDR score to indicate disorder.

### 3.3 Aggregation and amyloid predictions within the αSyn NAC alignment with 14-3-3.

Since disorder does not necessarily indicate the amyloidogenicity of protein, we used two methods to predict which residues are prone to aggregation. We first used PASTA 2.0 to determine a quantitative aggregation probability value for αSyn and the seven 14-3-3 isoforms. Fig. 2b shows a region of high aggregation probability between αSyn residues Val-63 to Ala-78, which is consistent with the experimentally defined NAC region of αSyn reported by Tuttle et al. in 2016 [56]. The 14-3-3 homologous residues Leu-12 to Gly-78 were overlaid with the αSyn sequence. We found a high aggregation probability for 14-3-3 residues Asn-42 to Val-52. This region shares a four-residue overlap with αSyn’s amyloidogenic region. PASTA 2.0 separately predicts parallel aggregation for αSyn between residues Val-63 to Val-82 (Fig. 1), which is again consistent with the crystallization data for αSyn amyloid fibrils [56]. Furthermore, PASTA 2.0 predicts parallel aggregation for six of the seven 14-3-3 isoforms (η, ε, β, σ, γ, and τ/θ) within the alignment region (the average residue length and location is also shown in Fig. 1). The overlap (αSyn residues Val-74-Val-82 and 14-3-3 residues Asn-43 to Val-52) is shown as a grey shaded region in Fig. 2b the length and location is also indicated by a yellow bar as in Fig. 1.

We then used the consensus method prediction tool AMYLPRED2 to further validate these regions of high amyloid propensity that are common to both αSyn and 14-3-3. AMYLPRED2 is a qualitative technique that uses an algorithm to determine if there is agreement in the aggregation predictions across 11 different methods to increase the confidence in the residue assignments [54]. Notably, AMYLPRED2 predicted amyloid aggregation behavior for αSyn residues Val-66 to Val-77 and 14-3-3 residues Leu-43 to Lys-49. Consistent with the PASTA2.0 predictions, we found these predictions correspond to a four-residue overlap between αSyn and 14-3-3 proteins (αSyn res Val-74 to Val-77). Since PASTA2.0 is not a subordinate method of the AMYLPRED2 algorithm, the agreement between the probabilities reported by both programs (Fig. 2) serves to increase the confidence of the predicted amyloid-prone regions of both proteins.

### 3.4 3D analysis of structural features in the αSyn tetramer and 14-3-3 dimer/tetramer within the BLAST NAC alignment region

**Fig. 4:**
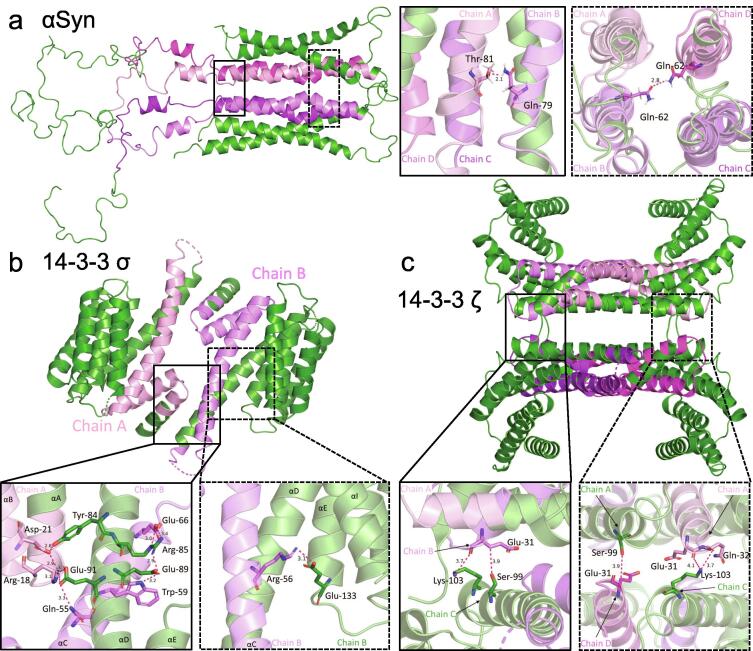
PyMOL 3D Structures of the 14-3-3 sigma dimer and the 14-3-3 zeta and αSyn tetramers. The alignment regions at αSyn residues Val-52 to Gly-111 are shown in shades of magenta: Chain A (pink), Chain B (violet), Chain C (light magenta), Chain D (purple). All structures are shown in *cartoon* representation, with individual residues in *stick* representation and bonds shown as magenta *dashed lines*. (a) Top panel: The NMR structure for the αSyn tetramer (Wang et al., 2011) as modified by Kara et al. The left inset (solid black boarder) shows Chain A Thr-81 hydrogen bond to Chain B Gln-79. The right inset (dashed black boarder) shows Chain B Gln-62 hydrogen bond to Chain D Gln-62. (b) Bottom left panel: The crystal structure of the human 14-3-3 sigma isoform (PDB ID:IYZ5), residues Leu-12 to Gly-78. The left inset (solid black boarder) shows amphipathic groove residues and homodimer contacts. The right inset (dashed black boarder) shows the Chain B intra-chain contact with the amphipathic groove residue Arg-56. (c) Bottom right panel: The crystal structure of human 14-3-3 zeta isoform (PDB ID:6EF5). The left inset (solid black boarder) shows the Chain B-Chain C inter-chain contacts. The right inset (dashed black line) shows the Chain A-Chain C and Chain A-Chain D inter-chain contacts. All images are generated using PyMOL molecular visualization software (pymol.org). Bond distances above are tabulated in the supplemental information. (For interpretation of the references to colour in this figure legend, the reader is referred to the web version of this article.)

We next extended our analysis to the 3D structures to significant residues at the dimer and tetramer interface of αSyn and 14-3-3 related to the sequence alignment. In this analysis, we focused on the longest alignment region between αSyn residues Val-52 to Gly-111 because the C-terminus is disordered. This region will be referenced as “the alignment region” for the remainder of this section. We used the 14-3-3σ X-ray crystallography coordinates (RCSB PDB ID: IYZ5) [38], the 14-3-3ζ/δ X-ray crystallography corrdinates (RCSB PDB ID:6EF5) and the NMR coordinates for the αSyn tetramer [21], [29], [30]. When we highlighted the alignment region in both proteins we found similarities in the characteristic secondary and quarternary structure in the folded proteins (Fig. 4). The αSyn alignment region is located in the core of the αSyn and 14-3-3 multimer (Fig. 4a). Here, we aimed to use the contact distances as a more quantitative way to guide our interpretation of which residues may be significant within the quarternary structures of both proteins.

First we identified hydrogen bonded contact residues in the αSyn tetramer. Although hydrogen bonds can be up to 3.5 Å, we choose the average hydrogen bonding distance of 3.0 Å as an upper limit to focus on the strongest interactors. Our area of interest is more expansive than a typical active site analysis, and the coordinates for αSyn were determined from an NMR structure instead of higher resolution crystallography coordinates (such as for 14-3-3 below). There were two contacts identified within the hydrophobic core of αSyn: (1) between the polar side chains of Thr-81 in Chain A and Gln-79 in Chain B (2.1 Å) and (2) a diagonal polar contact (2.8 Å) between the side chains of Gln-62 in Chain B and Gln-62 in Chain D. αSyn tetrameric structure exhibits intra-chain contacts between the hydrophobic inner helix (α2) and the outer n-terminal helix (α1) (Fig. S4.2). In Chain C, we found: (1) the residues Gln-79 and Glu-83 sidechains are within 1.8 Å of the charged residue, Lys-12, of the outer helix and (2) the residue Glu-57 sidechain interaction (1.8 Å) with Lys-34. These contact residues are critically located near the ends of the helices and the contact distances are within the expected range for hydrogen bonds.

Similarly, the 14-3-3 residues corresponding to the σ isoform alignment (Leu-12 to Gly-78) are located within the core of the 14-3-3σ dimer (Fig. 4b) and 14-3-3 tetramer (Fig. 4c) crystal structures. Here, we use a contact distance cutoff of 5.0 Å because binding residues and higher resolution crystal structures of 14-3-3 proteins have been studied in greater detail elsewhere [32], [38], [57]. This cutoff distance also allows us to exclude indirect interactions, and include weak, electrostatic interactions. The residues within the alignment region are consistent with the amphipathic groove as well as the dimerization contacts (Fig. 1). Residues within the alignment region that correspond to the amphipathic groove and may participate in the 14-3-3 phospho-serine/threonine motif binding include Leu-43, Lys-49, Arg-56. The 14-3-3σ Chain B residue Arg-56 side chain is within 3.1 Å of the residue Glu-133 as shown in the right inset for Fig. 4b, indicating the potential for Arg-56 to participate in stabilizing the dimerization of the sigma isoform. The dimerization residues Arg-18, Lys-74, Asp-21 are also found within the alignment region. Two of these residues Arg-18 and Asp-21, are identified in context of the residues Tyr-84 and Glu-91 and are shown in the left inset of Fig. 4b. It is generally accepted that the amphipathic groove is highly conserved across all 14-3-3 isoforms, and we also find the alignment region is very similar in structure forthe σ and ζ/δ isoforms.

In addition to the dimerization contacts, we found residues within the alignment region that may participate in the tetramerization of 14-3-3 during crystallization (Fig. 4c). On the right side of Fig. 4c, the Chain A residues Glu-31 and Gln-32 are 3.7 Å and 4.1 Å respectively of the charged amino acid Lys-103 in Chain C. Similarly, the Chain D residue, Glu-31 is 3.9 Å from Chain A residue Ser-99. The left side of Fig. 4c, the Chain B residue Glu-31 is 3.9 Å from the Chain C residue Ser-99 and 3.7 Å Lys-103. All interchain residues identified are within approximately 4.0 Å of the contact residues at the tetrameric interface for 14-3-3ζ/δ, indicating these contact distances are energetically significant and mostly electrostatic or weak interactions. The 14-3-3 tetramerization contact distances shown in Fig. 4 are shown in the supplemental information (Table S1.1). The analysis of contact residues is used to provide context for how residues within the alignment region may participate in structurally important interactions within the proteins.

### 3.5 STRING interaction networks predict functional similarity from verified interactions.

We used the STRING database to incorporate recent discoveries for PPIs with 14-3-3 and synuclein families. There is a much greater pool of data today than was known during the first homology analysis in 1999. The STRING database returned a total of 2947 interactions for the 14-3-3 family and 153 interactions for the synuclein family, all with a combined confidence score greater than 0.4. After comparing the two lists of interactions, we find 27 interactions, or node pairs, that are common to both protein families (Table 3). We define an interaction partner as a protein node connected by an edge to a member of the 14-3-3 family or to a member of the synuclein family, but not both. We define a shared interaction partner (SIP) as a protein connected by an edge to both 14-3-3 and to αSyn. The prediction scores for αSyn and 14-3-3 interactions showed a low to moderate significance for SIPs, with the highest sore being 0.525 for αSyn and 14-3-3η. The next highest scored SIP is for Calmodulin-1 (CALM1); the αSyn-CALM1 score is in the moderate range at 0.506 and the 14-3-3-CALM1 score is higher at 0.638. Fig. S5.1 in the supplemental information summarizes these results as an interaction network, where confidence scores are included for the top four scoring interactions with αSyn.

**Table 3:** STRING Database Network Results for αSyn matching node pairs to 14-3-3. The table is sorted by the αSyn combined score value. Node1 and node2 names are common to both 14-3-3 and the synuclein protein interaction network raw data tables from STRING. 14-3-3 final combined score is included in the last column to show matching score value to the αSyn combined score. The matching node pairs for proteins that interact with both αSyn and 14-3-3 are highlighted as: BAD (yellow), MAPT (green), TH (blue), and CALM1 (grey). Interactions between αSyn (SNCA) and 14-3-3 proteins (YWHAE, YWHAB, YWHAH) are denoted in red text. Double black lines are used to delineate a change in significance values for the combined score; >0.7, 0.5–0.7, and 0.4–0.5.

Our results in Table 3 show three of the SIPs have a combined score greater than 0.7 for at least one member of the protein family, indicating a high confidence in the prediction. These three proteins predicted to interact with both αSyn (SNCA) and 14-3-3 (YWHAB, YWHAH, YWHAE) are: microtubule-associated protein tau (MAPT), BCL2 associated agonist of cell death (BAD), and tyrosine hydroxylase (TH). Tau protein (MAPT) and BCL2 Associated Agonist Of Cell Death (BAD) resulted in the highest combined confidence score in both αSyn and 14-3-3 interactomes. Tyrosine hydroxylase (TH) resulted in a high confidence for αSyn interaction, and moderate confidence for 14-3-3 interaction. Importantly, these three SIPs showing the highest confidence scores are modulated by phosphorylation interactions [9], [58], [59]. This analysis confirmed that the same discoveries that have been shown previously remain important interactions, and can be identified using computational prediction methods.

## 4 Discussion

**Fig. 5:**
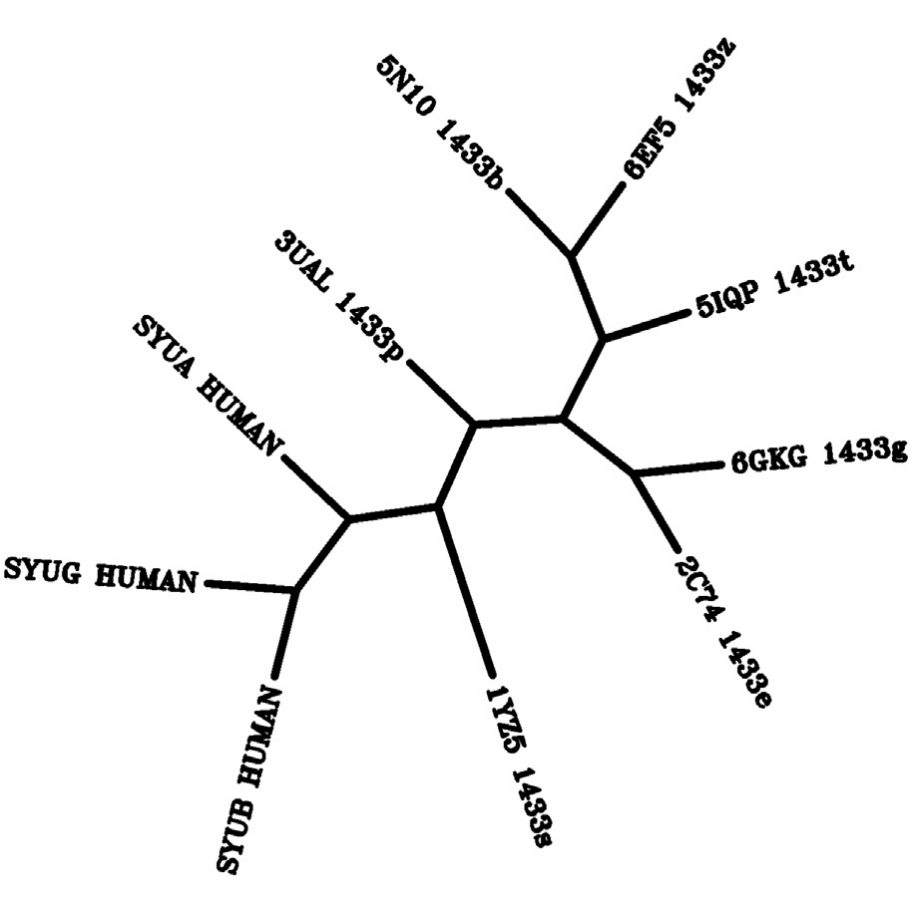
PHYLIP Phylogenetic tree of the synuclein and 14-3-3 proteins families. The phylogenetic tree shows the unrooted relative relatedness among the synucleins and seven 14-3-3 human isoforms according to the alignment illustrated in Fig. 1. Each branch is labeled according to the protein name for the synucleins and the RCSB PDB ID and protein name for the 14-3-3 proteins. The 14-3-3 labels are as follows: 14-3-3 sigma: “1YZ5_1433s”, 14-3-3 epsilon: “3UAL_1433p”, 14-3-3 eta: “2C74_1433e”, 14-3-3 gamma: “6GKG_1433g”, 14-3-3 theta/tau: “5IQP_1433t”, 14-3-3 zeta/delta: “6EF5_1433z”, 14-3-3 beta: “5N10_1433b”.

While a similarity between 14 and 3-3 proteins and αSyn had been identified previously [9], it was based on an MSA and no specific 14-3-3 isoform was highlighted. In contrast, we conducted pairwise alignments for each of the seven 14-3-3 isoforms. This choice was based on the fact that 14-3-3 isoforms exhibit characteristic structural and functional variations that are linked to the differences in isoform-specific direct contacts to their binding partners [41]. While this appraoch resulted in an alignment region in the 14-3-3 similar to the MSA-based one [9], the αSyn alignment regions are notably different. Specifically, there is alignment to the NAC, aggregation-prone region of αSyn, in addition to a short segment in the C-terminus of αSyn. Our comparison method allowed us to evaluate the alignment differences across isoforms, thus incorporate the potential significance of 14-3-3′s functional variation in our sequence alignment. This approach cast a spotlight on the 14-3-3σ isoform, as both sequence and disorder predictions indicated the 14-3-3σ isoform has the strongest homology to αSyn. This is highlighted in the evolutionary tree created based on our alignments that clearly positions the 14-3-3σ isoform in between the synuclein isoforms and the other 14-3-3 isoforms (Fig. 5). The alignment from 14 to 3-3σ residues Leu-11 to Gly-78 is unique to the σ isoform (Table 2), and the unique PONDR alignment in region 1 (Fig. 3) suggests there may be isoform-specific functional significance related to 14-3-3σ. It is particularly interesting to point out here that the 14-3-3σ isoform also shows a marked difference in Fig. 3 region 2 between Leu-100 to Tyr-130 and region 4 between Leu-170 to Asn-185 compared to the remaining six isoforms. The σ isoform is recognized to be unique among the 14-3-3 family; the first four helices exhibit sequence differences and the 14-3-3σ preferentially forms homodimers [41], [57]. There is also evidence suggesting isoform specificity of binding site, cell-type localization and cell development impact across the 14-3-3 isoforms [57], [60], [61]. Indeed, Yang et al. have extensively analyzed the structural flexibility of the 14-3-3 protein family, reporting that the binding interactions are dependent on isoform-specific structural features. This may be a surprising result, since 14-3-3σ is generally associated with tumorigenesis, and not with neurodegenerative disease. However, the distribution of isoform abundance in the brain was recently characterized for healthy subjects compared to patients with AD [62]. Gu et al. show an unequal distribution for the seven isoforms in the healthy brain samples as well as an unequal impact to the isoform expression in AD samples. 14-3-3 direct interactions with αSyn are also proposed to be isoform dependent, and 14-3-3σ isoform was discovered to colocalize with αSyn in Lewy Body neurites [50], [62]. αSyn's putative role in the gut microbiome is associated with the inflammatory response in the enteric nervous system and specifically in epithelial cells [12], [13], [63], [64]. This evidence may indicate a connection between αSyn-dependent PD and 14-3-3σ; both proteins are expressed in epithelial cells in addition to expression in neurons [57], [60]. These studies help to explain some of the variation in isoform-specific differences in the sequence alignment to αSyn. By consequence, our analysis may provide a clue to understanding the molecular variation of αSyn function and misfolding by focusing on the unique similarities to 14-3-3σ.

Experimental evidence identifies 14-3-3 proteins in Lewy Body neurites [65], and inclusion bodies with tau protein in Alzheimer’s disease [66], connecting 14-3-3 proteins with amyloidoses. The 14-3-3 proteins have a characteristic alpha-helical structure [41], [60], yet the NAC residues may be alpha-helical, or form the innermost core of the misfolded αSyn fibril [56]. Since the αSyn fibril forms beta-sheets and not alpha-helices, the similarity of these predictions may instead indicate a propensity for disorder-to-order transitions. This conclusion is supported by our disorder and amyloid predictions (for a detailed discussion, see supplementary discussion section). We expected to see a low probability of disorder for 14-3-3 in the PONDR results because the 14-3-3 dimeric and tetrameric crystal structures are reported as rigid, scaffold-like structures [34], [35]. Remarkably, PONDR predicted four of these regions to be disordered for the 14-3-3 isoforms (Fig. 3). While region 1 (N-terminal) and 5 (C-terminal) were highly similar across isoforms, regions 2, 3, and 4 were more variable. Region 1 showed significant long-range disorder and is consistent with the finding that there are dynamic properties in the amphipathic groove of 14-3-3 [41], [60]. The C-terminal tail is known to be dynamic, which is further evidenced by the absence of the C-terminal residues in the crystal structures [60]. Therefore, we reason the PONDR results correspond to the flexibility within the first four helices proposed to modulate binding interactions and the formation of hetero-dimers. A further comparison of the 14-3-3 PONDR data to the 14-3-3 PASTA2.0 and AMYLPRED2 results supports that the higher probability for aggregation is related to the contacts involved in dimer- and tetramerization. The overlapping regions of amyloidogenic propensity and structural disorder between αSyn and 14-3-3 correspond to αSyn residues Thr-72 to Val-82 (Figs. 2 and 3). This result is consistent across all seven human isoforms of 14-3-3 using the PASTA2.0 prediction (Fig. S4.1), suggesting this is a conserved feature. The disorder and amyloid prediction data corresponded well to the alignment predicted by BLAST alignment in the NAC region of αSyn (Fig. 2). We shaded five distinct regions in Fig. 3 to demarcate the 14-3-3 results. Thus, the unexpected prediction of long-range disorder at the N-terminus of 14-3-3 (14-3-3σ isoform residues Met-1 to Glu-75) may be key to understanding the amyloidogenic propensity of the NAC region in αSyn. To highlight this point further, although αSyn is known to misfold in the NAC region, it is also known to fold into an alpha-helical structure in the NAC region upon lipid binding [14], [27] and in the tetrameric species. This result established supporting evidence to the sequence homology between 14 and 3-3 and αSyn and suggests similarities in the disorder-to-order transitions of these proteins. We found only three proteins predicted to interact with both αSyn (SNCA) and 14-3-3 (YWHAB, YWHAH, YWHAE) with a >0.7 confidence score: microtubule-associated protein tau (MAPT), BCL2 associated agonist of cell death (BAD), and tyrosine hydroxylase (TH). The phosphorylation-dependent interactions with MAPT, BAD, and TH have been well-studied for both 14-3-3 proteins and αSyn [9], [58], [59], [67], [68]. Our STRING analysis did not reveal any additional shared interactions between αSyn and 14-3-3σ.

We also extended our analysis to the alpha-helically stabilized tetrameric species. We expected the amyloidogenic NAC region of αSyn to be within the central core of the tetramer based on previous experimental and computational reports [21], [28], [29], [30]. When we used the predicted sequence alignments from BLAST as a reference, we found corresponding similarity in the secondary and quarternary structure of 14-3-3 and αSyn (Fig. 4). The residue contacts for the 14-3-3σ dimer and the the 14-3-3ζ/δ tetramer interface (Fig. 4) are contained in the BLAST sequence homology illustrated in Fig. 1. Notably, the αSyn tetramer also seems to have stabilizing residues within the NAC region sequence alignment. Our analyses of the quaternary structures of αSyn and 14-3-3 further solidify our conclusion that αSyn has structural similarity to 14-3-3 and support the physiological relevence of the helical αSyn tetramer.

Overall, our results support αSyn function as a chaperone protein. There are two functions commonly ascribed to αSyn; (1) the lipid binding mechanism associated with facilitating neurotransmission [1], [14], [69] and (2) the participation as a molecular chaperone [1], [10], [11], [67]. 14-3-3′s chaperone behavior has been demonstrated experimentally [45] and the full-length HSPB6 dimer co-crystallizes in solution with dimeric 14-3-3σ binding within the canonical amphipathic groove. Their analysis shows a stabilization of the intrinsically disordered N terminal domain of pHSPB6 by 14-3-3σ [43]; a function that is seen in other 14-3-3 co-crystal structures as well [42], [43], [44]. The chaperone behavior of αSyn is experimentally linked to the NAC region [10], [67], and is shown to be abolished for a truncated peptide containing only residues Met-1 to Glu-61 [10]. This corresponds to related results that show C-terminally truncated αSyn increases aggregation [11], [67]. In fact, recent research shows a faster aggregation rate for αSyn C-terminal truncations shorter than Met-1 to Ser-129 [70]. Therefore, we propose that our sequence alignment segments corresponding to the C-terminus residues Ser-129 to Asp-135 and NAC residues Val-52 to Gly-111 are fundamental to the chaperone function of αSyn.

## 5 Conclusion

In order to develop an approach to treat PD and other neurodegenerative diseases involving αSyn misfolding, we need to better understand the structure and function of αSyn. This work provides an accessible methodological approach for analyzing inherited regions of intrinsically disordered proteins that may be structurally and functionally important, but not easily observed in vitro. In this paper, we identified corresponding sequence and structure similarities that supports the evidence that αSyn and 14-3-3 are homologs and that αSyn shares structural and domain architecture similar to chaperone proteins. The sequence homology between αSyn and 14-3-3σ suggested to us there may be functional specificity to the homology between 14 and 3-3 and alpha-synuclein that is isoform specific. This result highlights the importance of robust molecular level drug studies that consider the functional differences of proteins with highly similar isoforms. Our results linked αSyn's chaperone characteristics to the amyloidogenic NAC region, indicating a physiological basis for the self-assembly characteristics of the NAC region. The region of sequence homology found in the acidic c-terminal tail more strongly links αSyn to structural characteristics of chaperone proteins. Our results indicate there may be similarity in the domain architechture of αSyn and small heat shock protein chaperones that is distinct from the indirect neuroprotective effect attributed to αSyn's lipid binding mechanism. We explored the implications of our findings for the tetrameric species of αSyn and αSyn's proposed chaperone behavior, which indicates a molecular and evolutionary basis to support the physiological relevance of tetrameric αSyn. Our results may indicate a role for the αSyn tetrameric species as a molecular chaperone. We hope our results could be used to inform the contribution of αSyn's protein misfolding to the pathogenesis of neurodegeneration. Our results strengthen the physiological relevance of the αSyn tetramer and support the exploration of drug development that targets the stabilization of the ordered helical states of αSyn for the treatment of PD.

## CRediT authorship contribution statement

**Sarah R. Evans:** Investigation, Visualization, Methodology, Writing - original draft. **Colista West:** Investigation. **Judith Klein-Seetharaman:** Conceptualization, Funding acquisition, Investigation, Methodology, Project administration, Resources, Supervision, Writing - review & editing.

## Declaration of Competing Interest

The authors declare that they have no known competing financial interests or personal relationships that could have appeared to influence the work reported in this paper.
