# Supplementary material for: Similarity of the non-amyloid-β component and C-terminal tail of monomeric and tetrameric alpha-synuclein with 14-3-3 sigma
Source: Comput Struct Biotechnol J. 2021 Sep 14;19:5348–59. doi: 10.1016/j.csbj.2021.09.011 (PMC8495038; doi:10.1016/j.csbj.2021.09.011)
Supplement: Supplementary data 1 [file mmc1.docx]

Our pairwise sequence alignment approach allowed us to identify consensus alignments across multiple isoforms (**Table 1**). The isoform-specific alignments showed that the 14-3-3 alignment residues predicted by the BLAST alignment to αSyn are the same for the γ and η isoforms (**Table 1**). This result is consistent with previous analyses grouping these isoforms together [57]. There is similar structure-function variation in the synuclein family; most notably, βSyn is shown to be more resistant to aggregation than αSyn [1]. Thus, it is interesting that αSyn shares high sequence identity to four isoforms at the HI-loop (ζ/δ, ε, σ, β), since this region was shown to distinguish isoform-specific interactions [41,60]. Additionally, the loop predicted between αC and αD of 14-3-3σ and the C-terminal tail of αSyn (**Table 1**) appears homologous only in that these regions are similarly disordered. However, it is possible this region carries significance in more closely connecting αSyn to the σ isoform since the 14-3-3 αC-αD binding loop is most divergent in the σ isoform [41,57,60]. The τ/θ isoform does not show any significant sequence alignment to αSyn, even in the highly conserved HI-loop of 14-3-3. This result seems to be consistent with the observation that the τ/θ HI-loop is not involved in significant binding interactions [41,60]. These observations indicate the importance of evaluating individual protein family members in addition to the conserved properties of a protein family.

The disorder predictions indicate two distinct regions of the 14-3-3 isoforms that were not predicted to have disorder: region 2 from approximately Leu-100 to Tyr-130 and region 4 from approximately Leu-170 to Asn-185 (**Fig. 3**). In contrast to the σ isoform alignment region, regions 2 and 4 corresponded to an increased probability for aggregation. To explain this inconsistency, we referred to the training conditions of each algorithm. In particular, PASTA 2.0 assumes the native structure of a protein is disordered, which is not the case for 14-3-3. However, the PASTA 2.0 results are corroborated by the AMYLPRED2 prediction. If we then assess the PONDR results, an order prediction is consistent with disorder-to-order transition regions [52,53]. Both region 2 and 4 contain flexible loops, thus are likely to become more restricted upon dimer and tetramer formation. This interpretation is consistent with the reported characteristics of 14-3-3 proteins [34,35,60], suggesting regions 2 and 4 in the 14-3-3 proteins are likely to be prone to aggregation. Additionally, as the length of the region decreases, the liklihood of a false PONDR prediction increases; in both regions 2 and 4, the residue length is less than the ideal 39-residue stretch. Region 3 (residues 125-175) was predicted to be disordered, and includes αE and αF in the amphipathic binding groove. This result corresponds to the functional and structural adaptability observed among the 14-3-3 isoforms [41,60], and further associates regions of disorder-to-order transition in 14-3-3 proteins with amyloidogenicity. In addition, the c-terminus homology at αSyn residues Ser-129 to Asp-135 are predicted to be unstructured by PONDR in both proteins. Indeed, the crystal structures of 14-3-3 frequently show no density between helices αH and αI [34,60], supporting the prediction. This region is not predicted in our analysis to have high amyloidogenic characteristics for both αSyn and 14-3-3 (**Fig. 2**). Our data suggests that the flexible C-terminus in αSyn is a functional characteristic that cooresponds with the isoform-specific ligand discrimination directed by the HI loop in 14-3-3 proteins [60].

When we reviewed the synuclein family disorder predictions, we see the βSyn disorder prediction showed that the first peak is shifted left compared to αSyn. The peak of the βSyn disorder profile aligns with αSyn residue His-50; this residue is unique in the αSyn sequence from the uncharged Gln-50 to a charged His-50. The second region of disorder located at the C-terminus is similar to αSyn. The C-terminus regions of βSyn, γSyn and αSyn very closely overlap in magnitude and residue number near peak "2" (**Fig.** **S2.2**). The γSyn prediction shows the longest region of disorder in the synuclein family, with two small peaks that are centered at peak 1 (**Fig. S2.2**). This data indicates the functional differences among the human synucleins could be related to the disorder propensity, despite the high degree of sequence identity of the synucleins in the N-terminus (**Fig. S1.3**).

We hypothesized the use of a PPI database would benefit our analysis, since a substantial amount of research has expanded the understanding of 14-3-3 protein-interactions to now more than two thousand PPIs. The diversity of interactions modulated by the 14-3-3 protein family made it difficult to encompass a complete list of interaction partners related to αSyn. We therefore adapted a bioinformatics approach using the STRING database to computationally predict similarities between the synuclein and 14-3-3 interactomes. Using this method we found only three proteins predicted to interact with both αSyn (SNCA) and 14-3-3 (YWHAB, YWHAH, YWHAE) related to phosphorylation: microtubule-associated protein tau (MAPT), BCL2 associated agonist of cell death (BAD), tyrosine hydroxylase (TH). A model was proposed describing the potential regulatory effect of opposing interactions on TH by αSyn and 14-3-3 proteins in detail [58]. Similarly, the binding of αSyn and 14-3-3 proteins to BAD was shown to have an inverse relationship [9]. These proteins need to be phosphorylated to bind to 14-3-3, while αSyn binds to the dephosphorylated state [9,58]. Tau is also known to bind to both αSyn [59] and 14-3-3ζ/δ and β isoforms, but not γ and ε [68]. In contrast, CALM-1 binding interactions involving 14-3-3 and αSyn do not indicate the same phosphorylation-dependent relationship. While STRING did not expand our understanding of known interactions, our results indicate STRING is a valuable tool for quickly identifying notable interactions within large PPI networks.

Notably, STRING does not predict a shared interaction for protein kinase C (PKC), referenced by Ostrerova *et al*. We briefly looked into this result and found that Protein Kinase C is a family of serine-threonine specific protein kinases with distinct activation mechanisms and interactions. The Human Protein Database (HPRD) used in this analysis identifies that 14-3-3 interacts with multiple isoforms of Protein Kinase C (PKC) including: iota, delta, gamma, mu (alias D1), theta, alpha, and zeta. These interactions are isoform specific for the 14-3-3 proteins. HPRD annotates only PKC epsilon as an interaction partner for αSyn, and does not include PKC epsilon as an interactor for 14-3-3 proteins. We used GeneCards (<https://www.genecards.org>) to identify if there are missing interactions in the HPRD, and found there are annotations for PKC epsilon and delta in GeneCards that are not captured in the HPRD and vice versa.

We also note that HPRD does not include cysteine string protein-alpha (CSPα) as a shared interaction partner. The ability of αSyn to rescue neurodegeneration has been experimentally observed in response to the deficiency of the presynaptic chaperone, cysteine string protein-alpha (CSPα) [16]. This neuroprotective effect of αSyn is attributed to the indirect lipid binding interaction and not a direct PPI between αSyn and CSPα [16]. It is interesting to note that CSPα has been shown to undergo a distinct conformational change upon phosphorylation [70]. 14-3-3 proteins have been shown to bind directly to phosphorylated CSPα [71], which is consistent with phosphorylation-dependent binding mechanism discussed previously for 14-3-3 proteins. Notably, the 14-3-3σ isoform was not identified as a binding partner in this experiment [71].


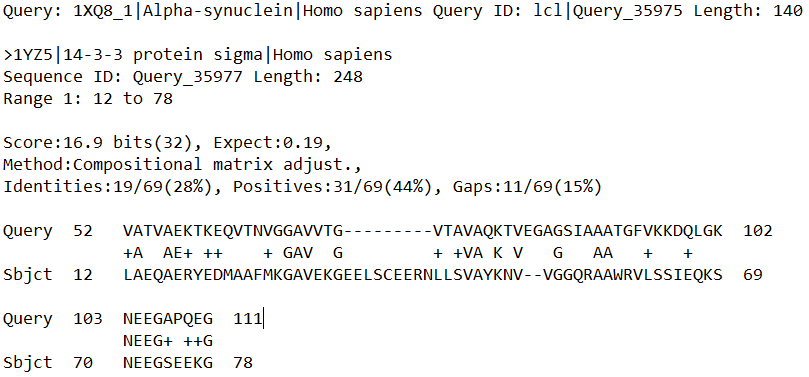


**Fig. S1.1 BLAST alignment output between αSyn and 14-3-3σ**. Image shows the raw data output for the BLAST sequence alignment between the query, αSyn, and the subject, 14-3-3σ. The nine gaps in the aSyn sequence that are hidden in Fig. 1 are shown. There are two alignment regions: Query residues 52-102, and 103-111.


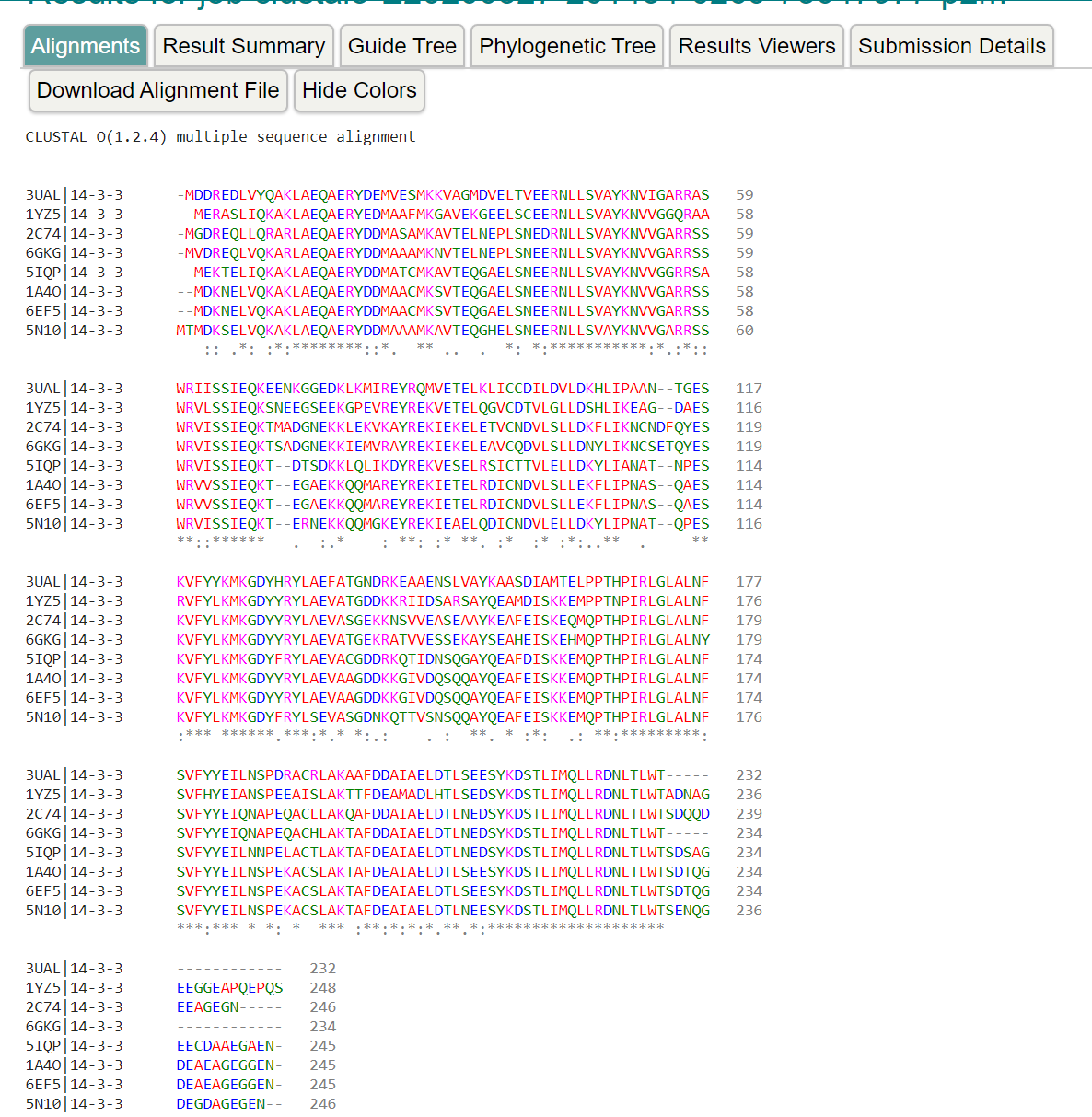


**Fig. S1.2 CLUSTAL Omega multiple sequence alignment for the human 14-3-3 protein family**. The multiple sequence alignment results are shown for the seven 14-3-3 isoforms found in the human genome. The bovin 14-3-3zeta isoform is also included as a control sequence since it has 100% identity with the human 14-3-3zeta isoform. From top to bottom the sequences are 14-3-3epsilon (3UAL), 14-3-3sigma (1YZ5), 14-3-3eta (2C74) 14-3-3gamma (6GKG), 14-3-3tau/theta (5IQP), bovin 14-3-3zeta (1A4O), 14-3-3zeta (6EF5), 14-3-3beta (5N10).


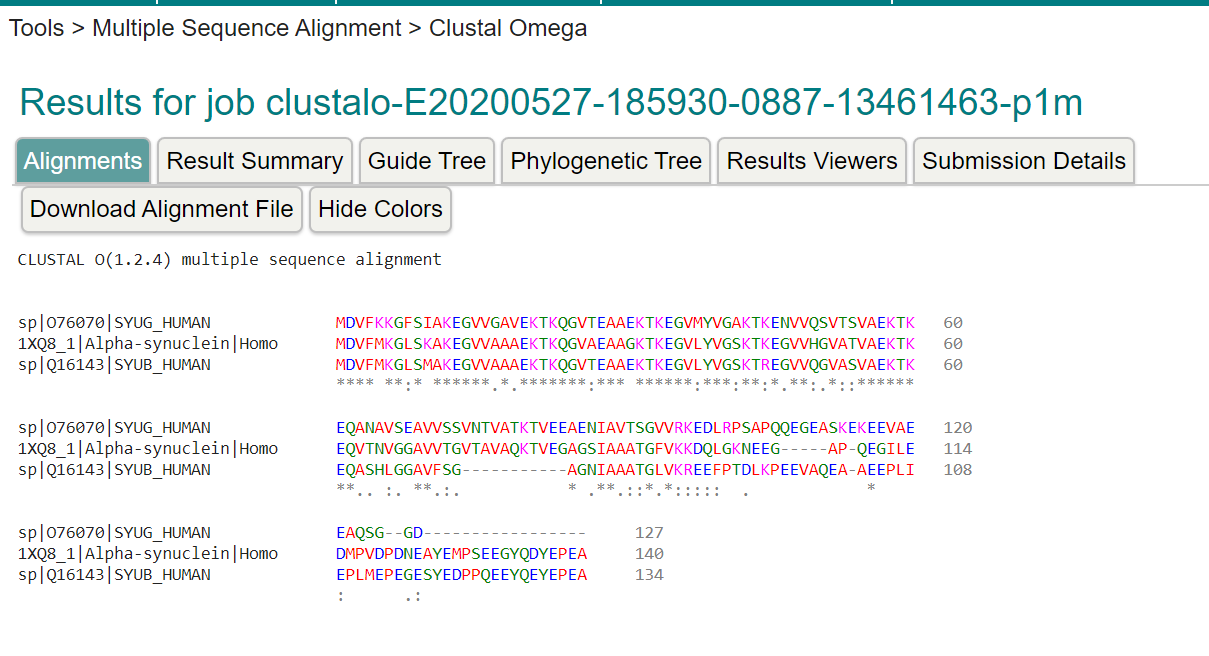


**Fig. S1.3 CLUSTAL Omega multiple sequence alignment for the synuclein Family**. The multiple sequence alignment results are shown for the synuclein family. From top to bottom the sequences are γSyn (SYUG_HUMAN), αSyn (Alpha-synuclein|Homo), βSyn (SYUB_HUMAN).

**
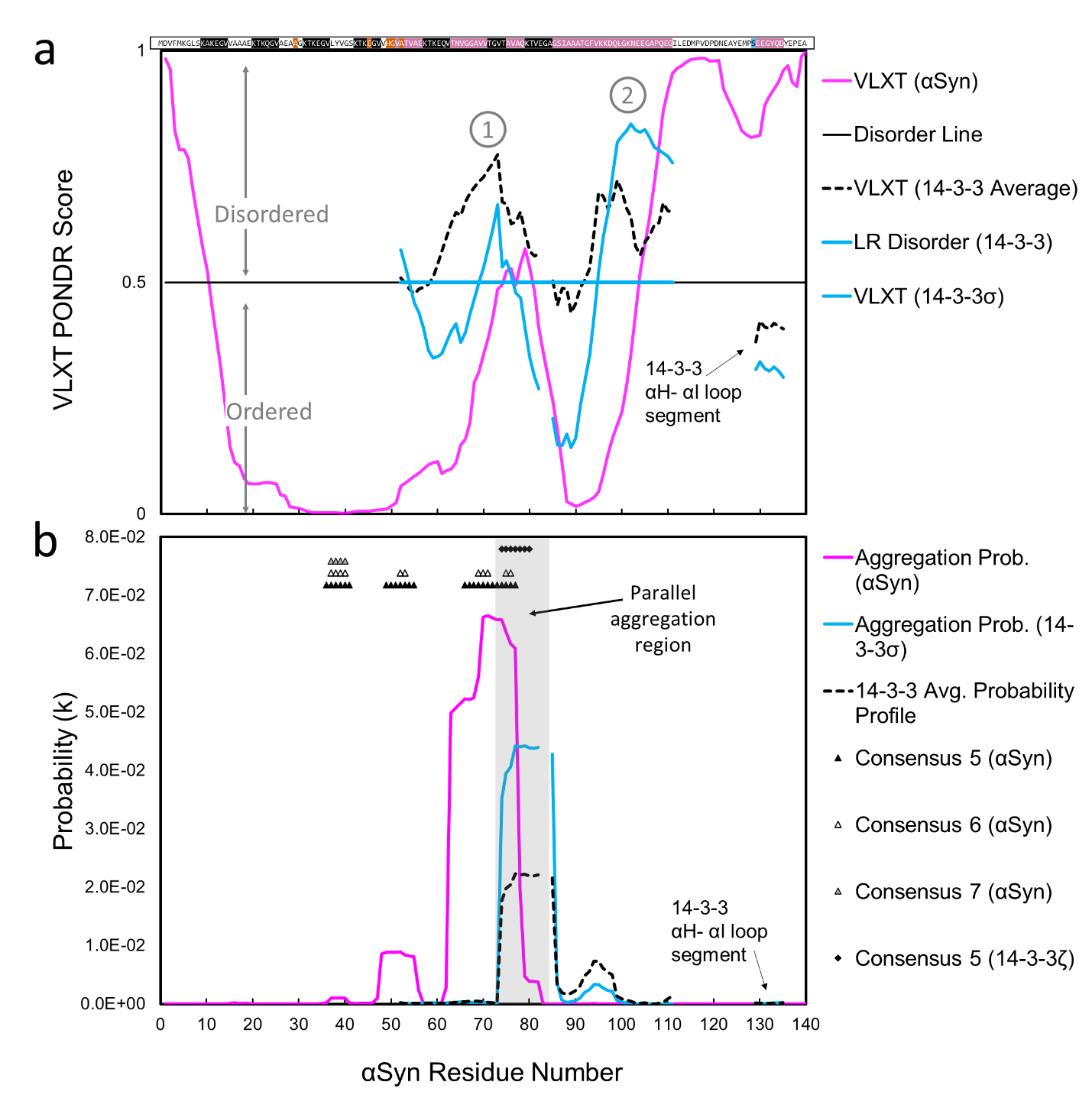
Fig. S2.1 Detailed Disorder and Amyloid predictions for αSyn overlaid with the 14-3-3 protein results**. (a) The VL-XT probability value is displayed on the y-axis plotted against the αSyn amino acid number on the x-axis. The disorder line is displayed at y-axis value of 0.5 (solid black line); the protein is predicted to be disordered for data above the line and ordered for data below the line. The VL-XT results for αSyn (solid magenta line), 14-3-3 sigma (solid blue line), and the averaged VL-XT data for all seven 14-3-3 isoforms (black dashed line) are shown with the αSyn amino acid number on the x-axis. The predicted long-range disorder for 14-3-3 is included as a thick solid blue line overlaid with the disorder line at y=0.5. Two distinct peak regions are indicated by the circled numbers 1 and 2 respectively. (b) The PASTA2.0 aggregation probability [k] results are shown on the y-axis plotted against the αSyn amino acid number on the x-axis; αSyn (solid magenta line), 14-3-3 sigma (solid blue line), and the averaged probability data for all seven 14-3-3 isoforms (black dashed line). The sequence alignment region of 14-3-3 is displayed against the corresponding αSyn residues Val-52 to Gly-111 and Ser-129 to Ala-135 predicted by BLAST. The AMYLPRED2 Consensus 5 predictions have no numerical value and are shown using a secondary axis as solid lines above the PASTA 2.0 results. The AMYLPRED2 results are colored as αSyn Consensus 5 (light blue), αSyn Consensus 6 (dark blue), αSyn Consensus 7 (light orange). The AMYLPRED2 Consensus 5 result for 14-3-3 zeta (dark orange) is displayed above the αSyn results. PASTA 2.0 predicts parallel aggregation for αSyn and 14-3-3 proteins. The common region is shown as a bold magenta line on the x-axis between residues Glu-72 and Arg-82. The aSyn sequence from Fig. 1 is shown at the top to show approximate alignments.

**
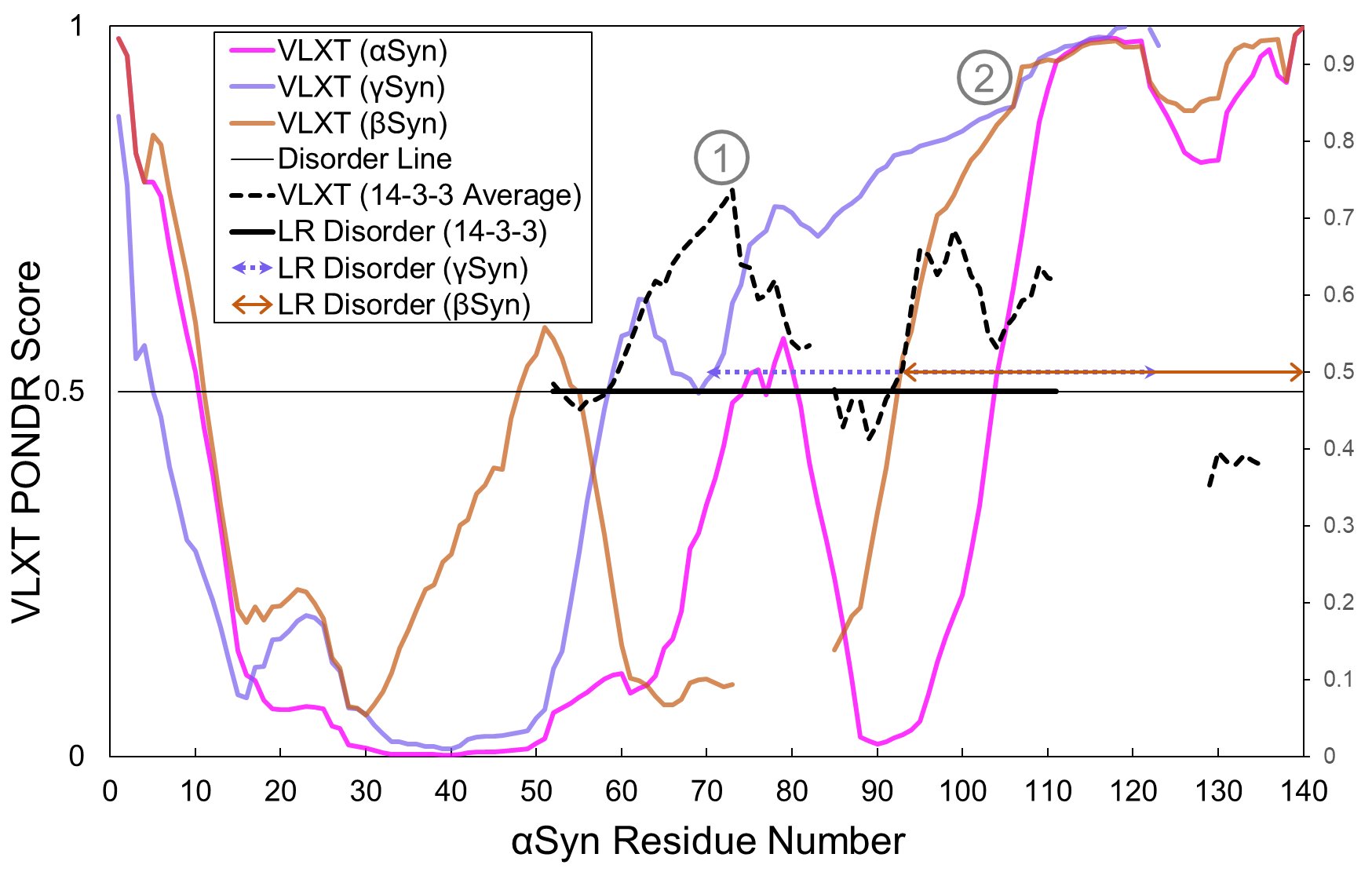
**

**Fig. S2.2 PONDR Score Overlay of αSyn, βSyn, γSyn, and 14-3-3 protein average VL-XT**. The VL-XT results for αSyn (solid black line), and the averaged VL-XT data for all seven 14-3-3 isoforms (black dashed line) are shown mapped to the aSyn sequence (x-axis scale). Similarly, the βSyn (magenta solid line) and γSyn (purple solid line) are shown mapped to their sequence alignment with αSyn. The predicted long-range disorder for 14-3-3 is included as a thick solid black line overlaid with the disorder line at y=0.5. The predicted long-range disorder for βSyn (magenta solid line with arrow end markers) and γSyn (purple solid line with arrow end markers) are shown using a second axis for clarity. The two distinct peaks in all data sets are indicated by the circled numbers 1 and 2 respectively.


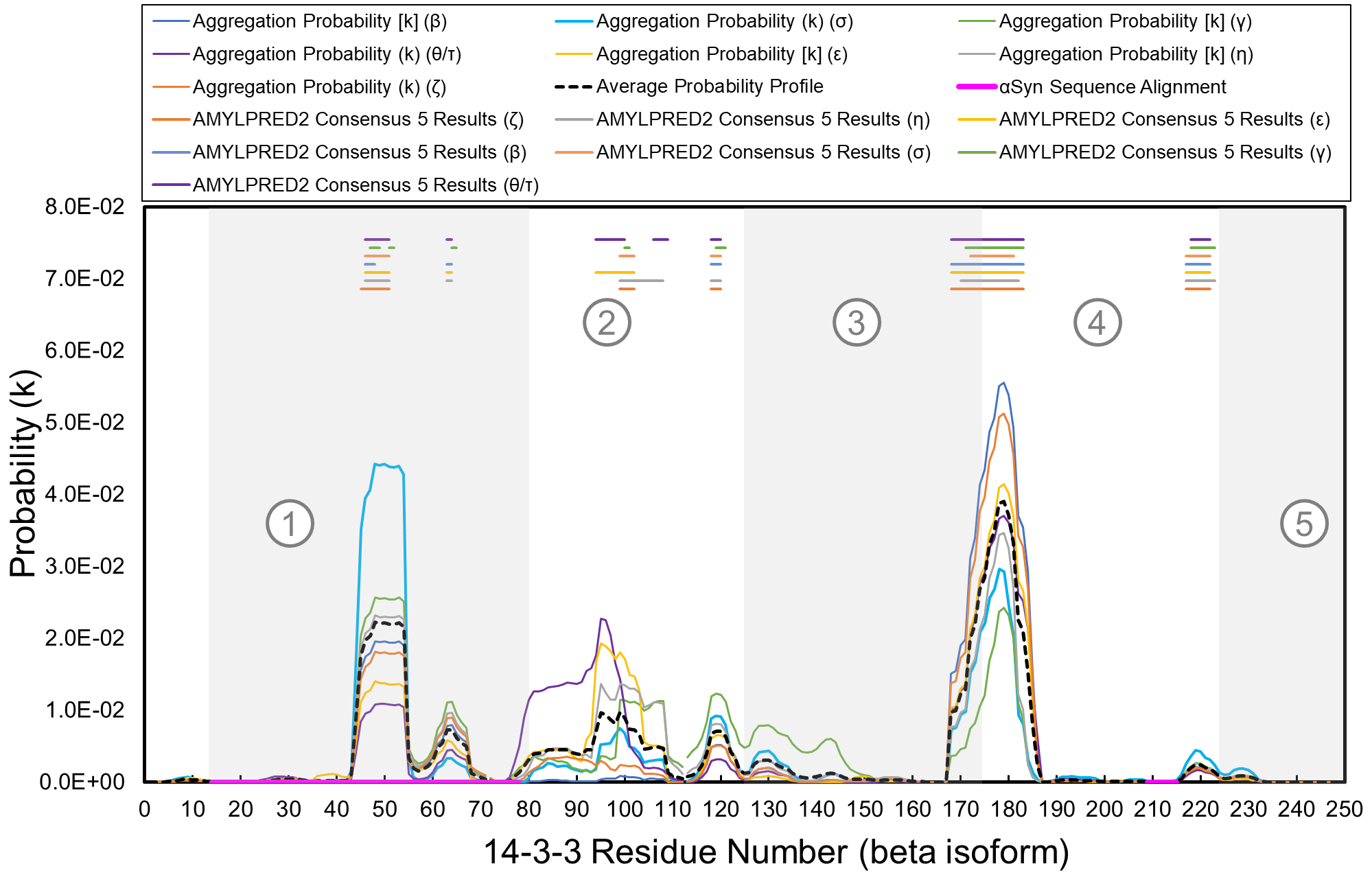


**Fig. S3.1** **PASTA 2.0 with AmylPred2 Prediction Overlay for 14-3-3 Protein family**. The PASTA2.0 aggregation probability for all seven human isoforms for 14-3-3 protein are overlaid together. The average probability is shown as a black dotted line. The sequence homology between alpha-synuclein NAC region (residues 52-111) and the c-terminus (residues 129-135) is represented at the base of the graph as a thick magenta line. The AMYLPRED2 Consensus 5 results are shown above the PASTA2.0 probability graph as straight lines indicating the residues involved in the prediction. Five distinct regions are identified with the numbers 1 through 5 from left to right with alternating shading. Region 1 contains residues from approximately 12-80; Region 2 contains residues 81-125; Region 3 contains residues from 126-175; Region 4 contains residues from 176-225; Region 5 contains residues from 226.


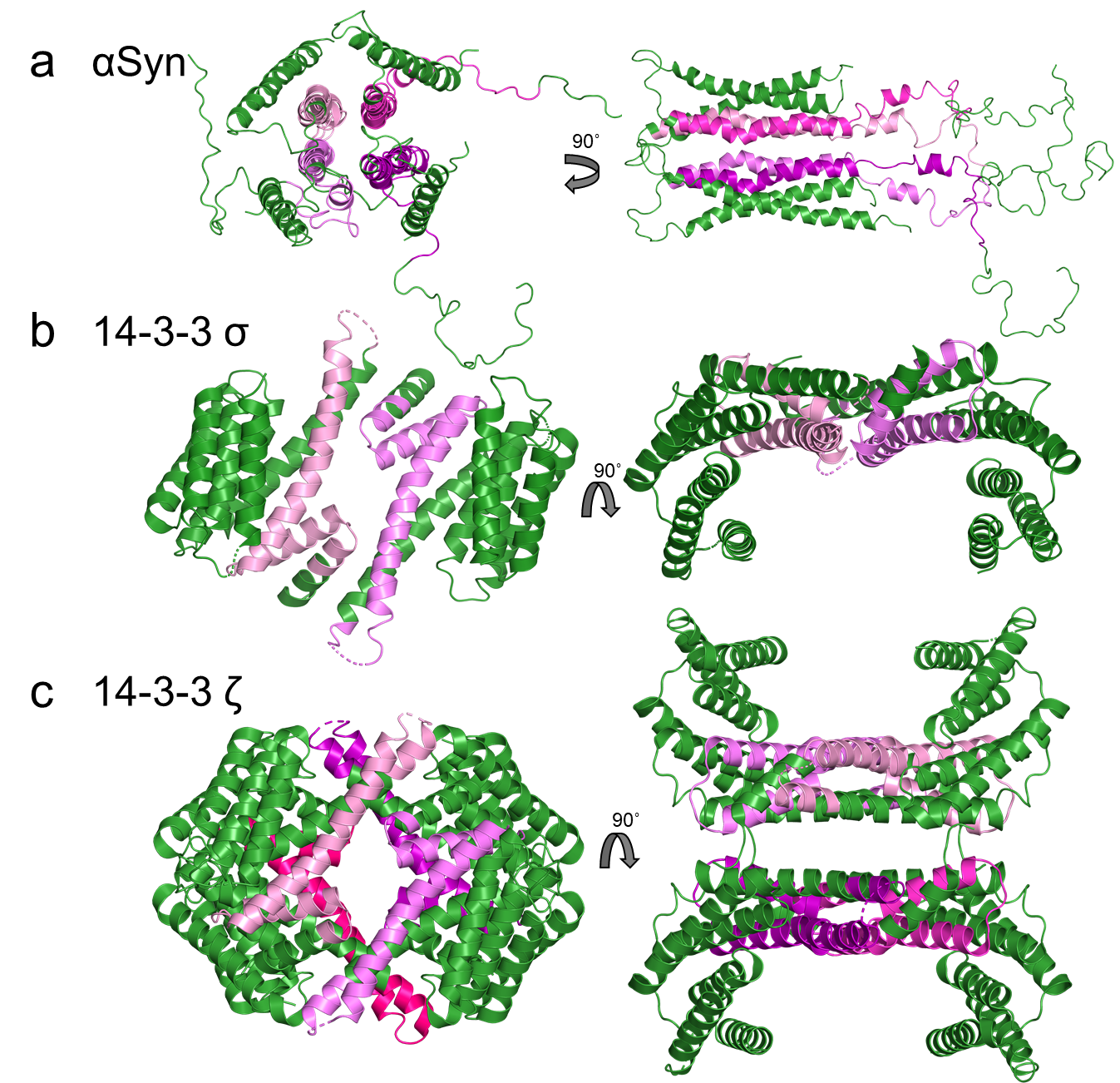


**Fig. S4.1 PyMOL images of "top" and "side" views of aSyn, 14-3-3sigma, and 14-3-3zeta**.

(a) The model structure for the αSyn tetramer (Wang et al., 2011) as modified by Kara *et al.* shown as *cartoon* representation in dark green. The homology segment containing residues Val-52 to Gly-111 is colored in magenta. The top-down view of the αSyn tetramer model structure is shown on the left and the side view of αSyn is shown after rotating 90 degrees into the plane of the page is on the right. (b) The crystal structure of the human 14-3-3σ isoform is shown in the top panel (PDB ID:IYZ5) shown as *cartoon* representation in dark green. The homology segment containing 14-3-3 residues Leu-12 to Gly-78 is colored in magenta. The top-down view of 14-3-3σ is shown on the left and the side view of 14-3-3σ after rotating 90 degrees into the plane of the page is shown on the right. (c) The crystal structure of human 14-3-3ζ isoform is shown in the middle panel (PDB ID:6EF5) shown as *cartoon* representation in dark green. The homology segment containing 14-3-3 residues Leu-12 to Gly-78 is colored in magenta. The top-down view of 14-3-3ζ structure is shown on the left and the side view of 14-3-3ζ after rotating 90 degrees into the plane of the page is shown on the right. Source of the αSyn tetramer structure coordinates: Courtesy of T. Pochapsky (Brandeis), John Hardy (UCL), and Patrick Lewis (RVC).


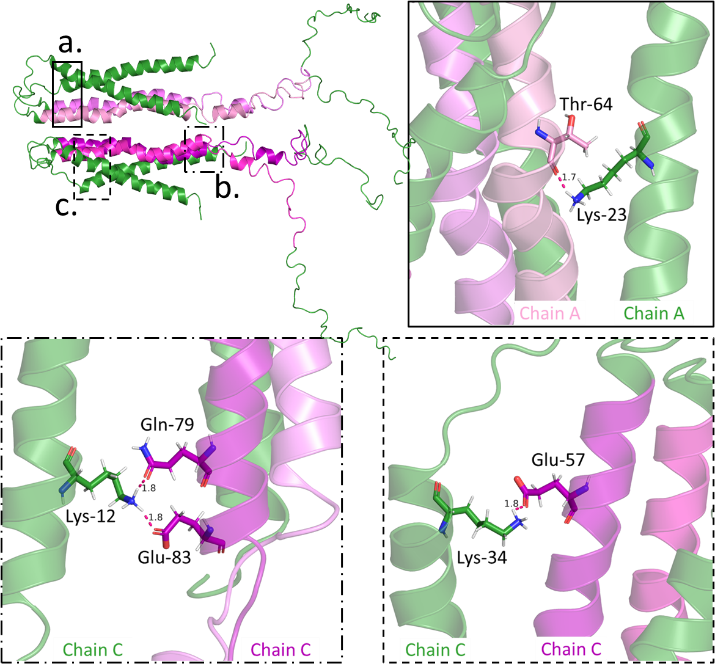


**Fig. S4.2 PyMOL images of αSyn Interchain Tetramer Contacts**. Detailed view of interchain residue contacts in the alpha synuclein tetramer. The structure is the same as in Fig.5 and is shown in *cartoon* representation, and the homology segment containing residues Val-52 to Gly-111 is colored by chain in shades of magenta. (a) Shows the polar contact between Chain A (Thr-81) and Chain B (Gln-79), and (b) the diagonal contact between Chain B (glutamine-62) and Chain D (glutamine-62) as a dashed purple line. Source of the αSyn tetramer structure coordinates: Courtesy of T. Pochapsky (Brandeis), John Hardy (UCL), and Patrick Lewis (RVC).

| Protein | Contact Description | Chain A | Chain B | Chain C | Chain D | Contact distance (Å) |
| --- | --- | --- | --- | --- | --- | --- |
| aSyn Tetramer contacts | *Intra-chain Contacts* |  | LYS-12,  GLN-79 |  |  | 1.8 |
|  |  |  | LYS-12,  GLU-83 |  |  | 1.8 |
|  |  |  | LYS-34,  GLU-57 |  |  | 1.8 |
|  |  | LYS-23,  THR-64 |  |  |  | 1.7 |
|  | *Inter-chain Contacts* | THR-81 | GLN-79 |  |  | 2.1 |
|  |  |  | GLN-62 |  | GLN-62 | 2.8 |
| 14-3-3σ Dimer Contacts | *Intra-chain Contacts* | ASP-21,  ARG-18 |  |  |  | 2.9 |
|  |  |  | GLU-66,  ARG-85 |  |  | 3.2* |
|  |  |  | ARG-85,  GLU-89 |  |  | 2.9 |
|  |  |  | GLU-89, T RP-59 |  |  | 3.2 |
|  |  |  | ARG-56,  GLU-133 |  |  | 3.1 |
|  |  |  | GLU-55,  GLU-91 |  |  | 3.3* |
|  | *Inter-chain Contacts* | ASP-21 | TYR-84 |  |  | 2.8 |
|  |  | ARG-18 | GLU-91 |  |  | 3.2 |
| 14-3-3ζ Tetramer Contacts | *Inter-chain Contacts* | GLN-32 |  | LYS-103 |  | 3.7 |
|  |  | GLU-31 |  | LYS-103 |  | 4.1 |
|  |  | SER-99 |  |  | GLU-31 | 3.9 |
|  |  |  |  | LYS-103 | GLU-31 | 3.7 |
|  |  |  |  | SER-99 | GLU-31 | 3.9 |

**Table S1.1 PyMOL 14-3-3 and αSyn Inter- and Intrachain Contacts**. Description of interchain residue contacts in the alpha synuclein tetramer, 14-3-3σ dimer, and 14-3-3ζ tetramer by chain. Contact residues formed within the same chain are contained in the same column, contact residues in different columns indicate the contact is formed between different chains. Contact distances marked with * are averages of two measurements.


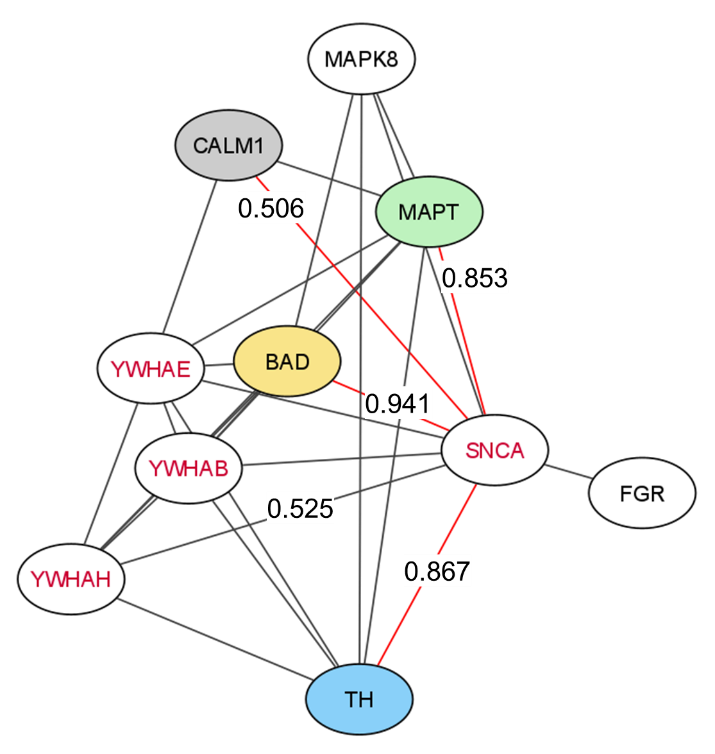
**Fig. 5.1 STRING database network common interactors for synuclein and 14-3-3 protein families**. The nodes for proteins that are found in the networks for both the synucleins and 14-3-3 proteins are shown in *ovals* identified by the protein *gene name* and connected by edges represented as *solid lines*. The proteins found to interact with both synucleins and 14-3-3 proteins are *highlighted* as: BAD (yellow), MAPT (green), TH (blue), CALM1 (grey). The nodes for αSyn (SNCA) and 14-3-3 proteins (YWHAE, YWHAB, YWHAH) are shown with red *text*. The edges connecting SNCA to the highest scoring interactors are shown as red solid lines. The *combined score* value for these interactions is overlaid with the respective edge. The interaction node pairs from Table 2 were used to generate an interaction network using Cytoscape software. The map is a *Force Directed* map created using the *combined score* values.
